# The TRIM69-MST2 signaling axis regulates centrosome dynamics and chromosome segregation

**DOI:** 10.1093/nar/gkad766

**Published:** 2023-09-22

**Authors:** Yilin Wang, Patrik Risteski, Yang Yang, Huan Chen, Gaith Droby, Andrea Walens, Deepika Jayaprakash, Melissa Troester, Laura Herring, Jonathan Chernoff, Iva M Tolić, Jessica Bowser, Cyrus Vaziri

**Affiliations:** Department of Pathology and Laboratory Medicine, University of North Carolina, Chapel Hill, NC 27599, USA; Division of Molecular Biology, Ruđer Boskovic Institute, Bijenicka cesta 54, 10000 Zagreb, Croatia; Department of Pathology and Laboratory Medicine, University of North Carolina, Chapel Hill, NC 27599, USA; Lineberger Comprehensive Cancer Center, University of North Carolina, Chapel Hill, NC 27599, USA; Joint Center for Single Cell Biology, School of Agriculture and Biology, Shanghai Jiao Tong University, Shanghai 200240, China; Department of Pathology and Laboratory Medicine, University of North Carolina, Chapel Hill, NC 27599, USA; Curriculum in Genetics and Molecular Biology, University of North Carolina, Chapel Hill, NC 27599, USA; Lineberger Comprehensive Cancer Center, University of North Carolina, Chapel Hill, NC 27599, USA; Department of Pathology and Laboratory Medicine, University of North Carolina, Chapel Hill, NC 27599, USA; Oral and Craniofacial Biomedicine Program, Adam’s School of Dentistry, University of North Carolina at Chapel Hill, NC 27599, USA; Department of Epidemiology, Gillings School of Global Public Health and UNC Lineberger Comprehensive Cancer Center, University of North Carolina, Chapel Hill, NC 27599, USA; Department of Pharmacology, UNC Proteomics Core Facility, University of North Carolina, Chapel Hill, NC 27599, USA; Fox Chase Cancer Center, Philadelphia, PA 19111, USA; Division of Molecular Biology, Ruđer Boskovic Institute, Bijenicka cesta 54, 10000 Zagreb, Croatia; Department of Pathology and Laboratory Medicine, University of North Carolina, Chapel Hill, NC 27599, USA; Department of Pathology and Laboratory Medicine, University of North Carolina, Chapel Hill, NC 27599, USA; Lineberger Comprehensive Cancer Center, University of North Carolina, Chapel Hill, NC 27599, USA

## Abstract

Stringent control of centrosome duplication and separation is important for preventing chromosome instability. Structural and numerical alterations in centrosomes are hallmarks of neoplastic cells and contribute to tumorigenesis. We show that a Centrosome Amplification 20 (CA20) gene signature is associated with high expression of the Tripartite Motif (TRIM) family member E3 ubiquitin ligase, TRIM69. TRIM69-ablation in cancer cells leads to centrosome scattering and chromosome segregation defects. We identify Serine/threonine-protein kinase 3 (MST2) as a new direct binding partner of TRIM69. TRIM69 redistributes MST2 to the perinuclear cytoskeleton, promotes its association with Polo-like kinase 1 (PLK1) and stimulates MST2 phosphorylation at S15 (a known PLK1 phosphorylation site that is critical for centrosome disjunction). TRIM69 also promotes microtubule bundling and centrosome segregation that requires PRC1 and DYNEIN. Taken together, we identify TRIM69 as a new proximal regulator of distinct signaling pathways that regulate centrosome dynamics and promote bipolar mitosis.

## Graphical Abstract

**Graphical Abstract:**
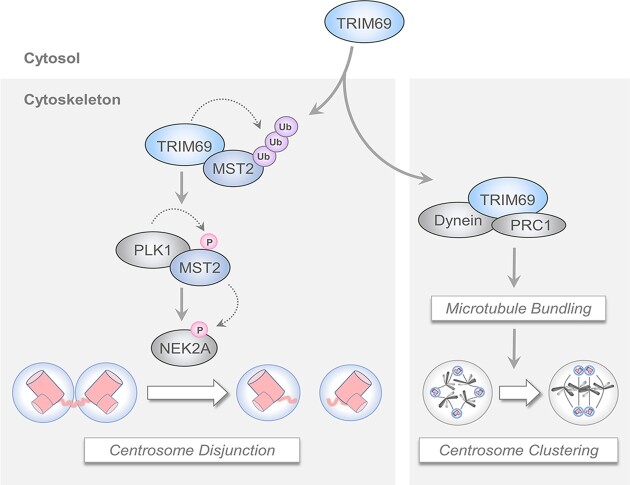

## INTRODUCTION

Accurate DNA replication and faithful/equal separation of replicated chromosomes into daughter cells is essential for maintaining genome stability and preventing cancer. The machinery that segregates the replicated chromosomes between daughter cells is termed the mitotic spindle (1). The spindle is comprised of two centrosomes (which serve as Microtubule-Organizing Centers or MTOCs), microtubules (MTs), and kinetochores. Beginning in prophase, each centrosome nucleates microtubules which either interdigitate with MTs from the other centrosome, or which attach to kinetochores (2). Some MTs emanating from the centrosomes also interact with factors at the cell cortex. The resulting MT network and associated factors collectively generate the forces that determine chromosome dynamics in mitotic cells.

Similar to DNA replication, centrosome duplication is tightly coupled/coordinated with the cell cycle and involves many of the protein kinases and ubiquitin ligases that regulate DNA synthesis. Polo-like kinase 4 (PLK4) is a master regulator of the centrosome cycle whose activity initiates centriole duplication (3,4). PLK4 overexpression alone leads to accumulation of multiple daughter centrioles adjacent to the mother centriole (3,4). PLK4 stability is tightly regulated by the Skp1–Cul1–F-box (SCF) complex SCF-bTrCP which restricts initiation of centriole duplication to a short time window at the G1/S transition (5,6). G2 cells normally contain two centrosomes, each comprised of two centrioles that are linked by rootletin, C-NAP1 and other proteins (5). Prior to mitosis the linker connecting centrioles is dissolved in a process termed disjunction. Disjunction is mediated by a protein kinase cascade initiated by PLK1 which phosphorylates and activates MST2/MST2. Activated MST2 phosphorylates and activates NIMA related kinase 2 (NEK2A), which in turn phosphorylates centrosomal protein 250 (C-NAP1), initiating untethering of the duplicated centrosomes (7). After disjunction, the centrosomes are separated and moved to opposite poles by the activity of the MT-dependent motor protein Eg5 (8). The force generated by Eg5-mediated sliding of antiparallel microtubules is sufficient to separate the centrosomes even when disjunction is impaired (7), demonstrating that the MST2-NEK2A and Eg5 pathways are redundant. Perturbation of disjunction and separation can severely compromise subsequent mitotic events. For example, incomplete spindle pole separation leads to higher rates of kinetochore mis-attachments, spindle multipolarity, chromosome mis-segregation and genomic instability (9).

Structural and numerical centrosome abnormalities are hallmarks of many cancers and are likely to contribute to genetic instability and tumorigenic phenotypes. Based on centrosomal abnormalities observed in early-stage tumors it has been suggested that altered centrosome biology may facilitate tumor initiation (10,11). Numerical alterations in centrosomes, most commonly in the form of centrosome amplification are frequently observed in cancer. Centrosome amplification has been observed in many solid and hematological cancer types and in many cancer cell lines (12). Notably, in a study of the NCI-60 panel, up to 62% of populations of lung cancer cell lines contained >4 centrioles. Studies in both flies and mammals show that centrosome amplification can be causally linked to aneuploidy and tumorigenesis (13,14). Centrosome amplification can arise via cytokinesis failure, mitotic slippage, cell–cell fusion, overduplication of centrioles and excessive de novo centriole assembly (10,11).

A 'Centrosome Amplification CA20' gene signature has been developed which comprises centrosome structural genes and genes that promote centrosome amplification such as polo-like kinase 4 (PLK4) (15). In a pan-cancer analysis of 9721 tumors in the TCGA, CA20 was associated with genomic instability, alteration of specific chromosomal arms, and poor prognosis (16). Notably, CA20 was highly associated with distinct clinical and molecular features of breast cancer.

The presence of two centrosomes at mitosis is required for a bipolar spindle, whilst excessive numbers of centrosomes lead to spindle multipolarity. Since many cancer cells harbor supernumerary centrosomes, they must adapt to withstand the presence of multipolar spindles. Four main processes are now known to avert lethal spindle multipolarity due to excess centrosomes: centrosome clustering (the best characterized mechanism in cancer cells), centrosome inactivation, centrosome degradation, and centrosome loss by extrusion (12).

The first molecule described to have a role in centrosome clustering was the minus-end-directed motor dynein (17). Subsequently, a screen for proteins involved in preventing spindle multipolarity in human cancer cells identified the chromosomal passenger complex, Ndc80 microtubule-kinetochore attachment complex, sister chromatid cohesion, and microtubule formation via the augmin complex as requirements for centrosomal clustering (18). Taken together, such studies indicate that factors controlling tension of the mitotic spindle apparatus are important for clustering of supernumerary centrosomes to form pseudo-bipolar spindles that support cytokinesis and viability (11). However, cells with pseudo-bipolar spindles fail to position all centrosomes appropriately on the bipolar axis and are prone to chromosome segregation defects (19). Thus, centrosome clustering in cancer cells may be a survival mechanism that also fuels further genome instability and drives tumor evolution.

Because centrosome clustering is necessary for viability of tumors harboring supernumerary centrosomes, disruption of centrosome clustering has been proposed as a potential therapeutic strategy in cancer (10,20). Lethal spindle multipolarity is also the mechanism of action of the antimitotic chemotherapeutic drug paclitaxel and clustering mechanisms confer resistance to this agent (21–23). Accordingly, there has been considerable interest in defining the centrosome clustering mechanisms deployed by neoplastic cells since these processes represent potential therapeutic targets.

In a preliminary analysis of TCGA data, we identified an association between the E3 ubiquitin ligase TRIM69 and centrosome amplification in cancer. Protein phosphorylation cascades and ubiquitin signaling events are jointly required for coordinating cell cycle events including centrosome duplication and movements (5,24–26). Therefore, we tested a hypothetical role for TRIM69 in regulating centrosome dynamics. Our results reveal a novel signaling pathway involving the protein kinase MST2 as a downstream effector of TRIM69 in a pathway that regulates centrosome disjunction. Additionally, we show that the TRIM69 promotes centrosome clustering through PRC1 and DYNEIN in cancer cells.

## MATERIALS AND METHODS

### Cell culture and transfection

Cancer cell lines MDA-MB-231, H1299, A549, U2OS and 293T were purchased from American Type Culture Collection (Manassas, VA, USA) and used for experiments without further authentication. All cell lines were cultured in Dulbecco's modified Eagle Medium (ThermoFisher, Waltham, MA, USA) supplemented with 10% fetal bovine serum and penicillin–streptomycin (1%). Plasmid DNA was transfected with Poly (ethyleneimine) (Sigma-Aldrich, St. Louis, MO, USA) and siRNA oligonucleotides were transfected using Lipofectamine™ 2000 Transfection Reagent (ThermoFisher) according to the manufacturer's instructions.

### Generation of stable cell lines

To generate the doxycycline (Dox)-inducible GFP-Plk4 U2OS cell lines stably expressing RFP-H2B, the cDNA fragment encoding GFP-Plk4 was PCR amplified from pEGFP-C3-PLK4-3xFLAG (Addgene, Cambridge, MA, USA) and subcloned into the pinducer20 plasmid, which placed it under transcriptional control of a doxycycline-regulated promoter. High-titer lentivirus was produced in HEK293T cells, U2OS cells were infected with lentivirus-containing medium containing 8 mg/ml polybrene (Sigma-Aldrich) in individual wells of a six-well plate. Medium was changed after 24 hours and stably-transduced cells were selected by growth in medium containing 1000 mg /ml G418 (ThermoFisher). Then the cells were infected with lentivirus expressing RFP-H2B and selected by growth in medium containing hygromycin B (ThermoFisher). To avoid clonal selection of idiosyncratic cells, pools of stably-infected cells were used for all experiments.

To generate *TRIM69A^−/−^* (‘TRIM69 KO’) lines, MDA-MB-231 cells were infected with pLENTI CRISPR-derived lentivirus (encoding sgRNAs and CAS9) produced in HEK293T cells. Stably-transduced cells were selected in 1 mg /mL puromycin for 7 days. Single colonies were selected using cloning cylinders (Corning) and genome editing was confirmed using the TIDE assay (27) (Supplementary Figure S3).

To reconstitute CAS9-resistant TRIM69A in MDA-MB-231 *TRIM69A^−/−^* cells, the PAM sequences of wild-type and E3 ligase-mutant TRIM69A cDNAs (corresponding to sites targeted by TRIM69A sgRNAs in the knockout cell line) were mutated to synonymous codons. *TRIM69A* cDNAs harbouring silent mutations were subcloned into pINDUCER20 and the resulting lentiviral vector was packaged to generate high-titer virus. MDA-MB-231 *TRIM69A^−/-^*cells were infected with pINDUCER-20 TRIM69A lentiviruses. Stably-transduced cells were selected in growth medium containing 1000 mg/ml G418 (ThermoFisher). Doxycycline-inducible reconstitution of TRIM69A expression was validated by SDS-PAGE and immunoblotting.

### Plasmid construction

pcDNA-HA-MST2 was a gift from Kunliang Guan (28) (Addgene plasmid # 33098), pEGFP C3-Mst2 was a gift from Marius Sudol (29) (Addgene plasmid # 19056), pEGFP-C3-PLK4-3xFLAG was a gift from Michel Bornens (30) (Addgene plasmid # 69837), pEGFP Centrin2 (3) (Nigg UK185) was a gift from Erich Nigg (Addgene plasmid # 41147), 8xGTIIC-luciferase was a gift from Stefano Piccolo (31) (Addgene plasmid # 34615), pRK5-HA-Ubiquitin-K63, pRK5-HA-Ubiquitin-K33, pRK5-HA-Ubiquitin-K48, pRK5-HA-Ubiquitin-K27, pRK5-HA-Ubiquitin-K29, pRK5-HA-Ubiquitin-K11, pRK5-HA-Ubiquitin-K6, pRK5-HA-Ubiquitin-WT, pRK5-HA-Ubiquitin-KO were gifts from Ted Dawson (32) (Addgene plasmid #17606, #17607, #17605, # 22902, #22903, #22901, #22900, #17608, #17603). pLKO-RFP-H2B plasmid was kindly provided by Dr Williams Scott E. mCherry-PRC1 plasmid was obtained from Casper C. Hoogenraad (33) . Myc- TRIM69A was a gift from Dr Angelique Whitehurst (UT Southwestern) and Myc- TRIM69B was PCR amplified from Myc- TRIM69A and constructed in pcDNA3 vector. pEGFP-MST2 and HA-MST2 site mutants were derived from pEGFP-C3-MST2 and pcDNA-HA-MST2 by PCR using conventional methods respectively. To generate the HA-TRIM69A and Flag-TRIM69A vectors, the TRIM69A open reading frame was PCR amplified from pcDNA3 Myc-TRIM69A plasmid and subcloned into the pcDNA3 expression plasmids. TRIM69A mutants harboring internal deletions and individual nucleotide substitutions were derived by PCR using conventional methods. NEK2A was PCR amplified from the cDNA and subcloned to the pcDNA3 plasmid. The full length region of TRIM69A, TRIM69B, MST2 and MST1 were cloned from pcDNA3-Myc- TRIM69A, pcDNA3.1-Myc-TRIM69B, pcDNA-HA-MST2 and pJ3H-MST1 plasmids and then fused to either N-terminal GAL4 DNA activation domain (AD) in the pDEST-GADT7 vector or N-terminal GAL4 DNA binding domain (DBD) in pDEST-GBKT7. The lentiCRISPR v2 vector (Addgene) expressing Cas9 and containing a cloning site for the sgRNA sequence was digested with BsmBI (NEB, Ipswich, MA, USA). The TRIM69A sgRNA-1, -2 were synthesized, annealed, and ligated to the lentiCRISPR v2 plasmid. Sequences of gene-specific primers and designed sgRNAs used in this study are listed in Supplementary Table S1. The amplified fragments by PCR in all constructs and insertion of the sgRNA were validated by DNA sequencing.

### Yeast two-hybrid assay

The yeast two-hybrid assay was carried out as described previously (34). The full length coding sequences of TRIM69A, TRIM69B, MST2 and MST1 were fused to either N-terminal GAL4 DNA activation domain (AD) in the pDEST-GADT7 vector or N-terminal GAL4 DNA binding domain (DBD) in pDEST-GBKT7. The Saccharomyces cerevisiae yeast strain Y187 transformed with GAL4-DBD fusion protein was mated with the yeast strain AH109 transformed with GAL4-AD fusion protein. The pDEST-GADT7-GUS construct was used as a negative control. The fresh diploids on the double dropout (DDO) medium were placed on selective triple dropout medium (TDO, without Leu, Trp and His) plus 1 mM 3-aminotriazole (3-AT) and quadruple dropout (QDO) medium (without Leu, Trp, His and Ade), plates were incubated at 30°C for 3 days before been collected.

### Adenovirus construction and infection

TRIM69A, TRIM69B and Control adenoviruses were constructed and purified as described previously (35). In brief, cDNAs encoding TRIM69A, TRIM69B were subcloned into the pACCMV shuttle vector. The resulting shuttle vectors were co-transfected with the pJM17 adenovirus plasmid into HEK293T cells. Recombinant adenovirus clones were isolated by plaque purification and verified by restriction analysis and Southern blotting. The empty vector Ad-Control (used to control for adenovirus infections) was derived similarly but by co-transfection of the parental pACCMV shuttle vector with pJM17. Adenovirus particles were purified from 293T cell lysates by polyethylene glycol precipitation, CsCl gradient centrifugation, and gel filtration column chromatography. Adenovirus preparations were quantified by A260 measurements. Cells were typically infected with 0.1−1.0 × 10^10^ pfu/ml by direct addition of purified virus to the culture medium.

### RNA interference

For H1299, MDA-MB-231and U2OS cell lines, siRNAs were reverse-transfected using Lipofectamine 2000. In brief, siRNAs were incubated with Lipofectamine 2000 and serum-free OptiMEM for 15 min at room temperature in the dark. Cells were then trypsinized and resuspended in 1 ml of OptiMEM and added directly into the siRNA/OptiMEM/Lipofectamine solution to give a plating density of 50%, and then they were incubated for 48 h. The siRNA sequences were listed in Supplementary Table S1.

### Immunofluorescence and live cell microscopy

H1299, U2OS and MDA-MB-231 TRIM69A KO cells were grown on coverslips and fixed for 15 min in 2% formaldehyde, rinsed 3 times in PBS, then permeabilized in PBS–0.1% NP40. Cells were blocked in 2% BSA for 10 min and probed with primary antibodies listed below for 1 h. Coverslips were rinsed 3 times in PBS and probed with Alexa-conjugated secondary antibodies for 1 hour. Coverslips were then rinsed 3 times in PBS and coverslips were mounted using Fluoro-Gel (Electron Microscopy Sciences, Hatfield, PA, USA) mixed with DAPI. Primary antibodies including: Mouse Anti-Human Pericentrin Monoclonal Antibody, Unconjugated (Abcam, ab28144, Cambridge, UK), Rabbit Anti-Pericentrin Polyclonal Antibody, Unconjugated (Abcam, ab4448), β Tubulin antibody—Loading Control (Abcam, ab6046), HA tag antibody—ChIP Grade (Abcam, ab9110), Mouse Anti-HA tag Monoclonal Antibody, Unconjugated, Clone HA.C5 (Abcam, ab18181), c-Myc antibody [9E10] - ChIP Grade (Abcam, ab32), Nuclear Pore Complex Proteins antibody [Mab414]—ChIP Grade (Abcam, ab24609), DYNLL1 antibody (Abcam, ab51603), Anti-PLK1 antibody (Abcam, ab189139), C-NAP1 antibody (Proteintech,14498–1-AP, Rosemont, IL ), Acetylated-α-Tubulin (Santa Cruz Biotechnology, sc-23950, Dallas, TX ), PRC1 (Invitrogen, PA5-30296, Waltham, MA).

For image acquisition, we used an Andor Dragonfly Spinning Disk Confocal Microscope (OXFORD Instruments America, Concord, MA) mounted on a Leica DMi8 microscope stand, equipped with an HC PL APO 100×/1.40 OIL CS2 Leica objective. The pinhole size was set to 40 μm. The camera was a Zyla Plus 4.2MP sCMOS with 2048 × 2048 pixels, with an effective pixel size of 0.063 um. A piezoelectric Z stage was used to acquire Z stacks at 0.147 um intervals. Z stack size ranged between x and y um. Excitation lasers were 405 nm (for DAPI), 488 nm (for AlexaFluor 488) and 561 nm (for AlexaFluor 594). Emission filters were 445/46 (for DAPI), 521/38 (for AlexaFluor 488) and 594/43 (for AlexaFluor 594). Images were deconvolved in Autoquant.

H1299 cell lines stably expressing RFP-H2B or Dox-inducible GFP-PLK4 U2OS cell lines with stable RFP-H2B expression were seeded on Chambered Coverglass from Lab-Tek II (ThermoFisher, 155382). H1299 RFP-H2B cell lines were transfected with siRNA for 48 h before exposed to paclitaxel for 24 h while U2OS cell lines induced PLK4 expression 24 h post transfection. Time-lapse microscopy was performed on a Keyence BZ-X810 using a 40× objective. Images were taken at 2 min interval for 24 h. Best focus projections of the time series were exported into AVI format. Image sequences were generated using ImageJ and manually quantified.

### Image analysis

Quantification of the spindle tubulin intensity was done on a sum-intensity projection of all z-planes in which the spindle was positioned. We used the Polygon selections tool in Fiji/ImageJ (National Institutes of Health, Bethesda, MD, USA) to encompass the area of the spindle and measure the mean spindle intensity. Mean spindle intensity was background corrected by subtracting the mean intensity of the cytoplasm and normalized by dividing it by the number of z-planes in which the spindle was positioned. Spindle PRC1 intensity was quantified in the same manner.

By using the Line tool in Fiji/ImageJ, the tubulin signal intensity of a cross-section of an interphase bundle was measured by drawing a 5-pixel-thick line perpendicular to the tubulin signal. The tubulin intensity profile was corrected by subtracting the mean background from the cytoplasm. The signal intensity of the interphase bundle was calculated as the area under the peak using SciDavis (Free Software Foundation Inc, Boston, MA, USA). The same tubulin intensity profiles were used to calculate the thickness of interphase bundles, i.e. by measuring the width at the base of the tubulin signal intensity peak. Five bundles per inspected cell were analyzed. Quantification of the colocalization was done using ImageJ plug-in JAcoP.

### RNA extraction, reverse transcription and real-time PCR

RNA samples were extracted with RNeasy Mini Kit (QIAGEN, Valencia, CA, USA). Reverse transcription assay was performed by using the iScript cDNA Synthesis Kit (BIO-RAD, Hercules, CA, USA) according to the manufacturer's instructions. Real-time PCR was performed by using iTaq Universal SYBR Green Supermix (BIO-RAD). For quantification of gene expression, the 2^–ΔΔCt^ method was used. GAPDH expression was used for normalization. The sequence information for each primer used for gene expression analysis was listed in the Supplementary Table S1.

### IP and immunoblotting

Immunoprecipitation and immunoblotting methods were carried out as described (36). Briefly, to prepare cell extracts containing soluble and CSK-insoluble nuclei, monolayers of cultured cells were washed twice in ice-cold PBS and lysed in ice-cold cytoskeleton buffer (CSK buffer: 10 mM Pipes, pH 6.8, 100 mM NaCl, 300 mM sucrose, 3 mM MgCl_2_, 1 mM EGTA, 1 mM dithiothreitol, 0.1 mM ATP, 1 mM Na_3_VO_4_, 10 mM NaF and 0.1% Triton X-100) freshly supplemented with cOmplete protease inhibitor cocktail (Roche, Indianapolis, IN, USA) and PhosSTOP (Roche). Lysates were centrifuged at 4000 rpm for 4 min to remove the CSK-insoluble nuclei. The detergent-insoluble nuclear fractions were washed once with 1 ml of CSK buffer and then resuspended in a minimal volume of CSK, then sonication was performed followed by nuclease treatment. For whole cell lysate, soluble and CSK-insoluble fraction were combined before analysis by SDS-PAGE and immunoblotting. For immunoprecipitation (IP) experiments, magnetic beads containing covalently conjugated antibodies against the HA tag, Myc tag or Flag tag (MBL international Corporation, Woburn, MA, USA) were added to the extracts, and incubations were performed for 3 h at 4°C using rotating racks. Immune complexes were recovered using magnetic stands. The beads were washed five times with 1 ml CSK (1 min per wash) to remove nonspecifically associated proteins. The washed immune complexes were boiled in protein loading buffer for 10 min to release and denature for SDS-PAGE. For immunoblotting, cell extracts or immunoprecipitates were separated by SDS-PAGE (for Phos-tag phosphate affinity gel electrophoresis, SuperSep Phos-tag (50μmol/l), 7.5%, 17-well, 100 × 100 × 6.6 mm gel from FUJIFILM Wako Chemicals was used), transferred to nitrocellulose membranes, and incubated overnight with the primary antibodies at manufacturer's recommended concentrations listed below: GAPDH (6C5) antibody (Santa Cruz Biotech, sc-32233), Anti-Histone H3 antibody (Abcam, ab176842), MST2 antibody (Abcam, ab52641), MST1 antibody [EP1465Y] (Abcam, ab51134), Anti-phospho-Histone H2A.X (Ser139) Antibody, clone JBW301 (EMD Millipore, 05-636), Anti-PLK1 antibody ( Abcam, ab189139), HAUS1 Monoclonal Antibody (ThermoFisher, MA5-22937), Phospho-YAP (Ser397) (Cell Signalling Technology, 13619S, Danvers, MA, USA), YAP Antibody (Cell Signalling Technology, 4912S), YAP1 (phospho S127) antibody (Abcam, ab76252), p-Cdk (Thr14/Tyr15)-R antibody ( Santa Cruz Biotech, sc-28435-R), MST1/MST2 (phospho T183 + T180) antibody (Abcam, ab76323), Phospho-MOB1 (Thr35) (D2F10) Rabbit mAb antibody (Cell Signalling Technology, 8699S), Rabbit Anti-Human Cyclin E (C-19) Polyclonal, Unconjugated antibody (Santa Cruz Biotech, sc-198), Anti-SAV1 antibody (Abcam, ab230265), Recombinant Anti-NUP133 antibody (Abcam, ab155990), Anti-Nup107 antibody (Abcam, ab178399), Recombinant Anti-Eg5 antibody (Abcam, ab254299), Dynein Monoclonal Antibody (Invitrogen, MA1-070), DYNLL1 antibody (Abcam, ab51603), Recombinant Anti-CENPF antibody (Abcam, ab223847), NEK2 Polyclonal antibody (Proteintech, 14233–1-AP), DYKDDDDK Tag (D6W5B) Rabbit mAb (Cell Signalling, 14793S), Anti-alpha Tubulin antibody (Abcam, ab4074). Phospho-MST2 (Ser316) Polyclonal Antibody (ThermoFisher, PA5-105065). Then one hour incubation with the secondary antibodies in 5% nonfat milk TBST. Perkin Elmer Western Lightning Plus ECL was used to develop films.

### Ubiquitination Assay

HA-MST2, Myc-TRIM69A and Myc-TRIM69A E3 mut were transfected into 293T cells together with or without Flag-Ub. The cells were then treated with MG132 (20 μM) for 8 h and lysed by RIPA buffer with Protease Inhibitor Cocktail and Phostop (Roche). Magnetic beads containing covalently conjugated antibodies against the HA tag, Myc tag or Flag tag (MBL international Corporation) were added to the extracts, and incubations were performed for 3 h at 4°C using rotating racks. Then the samples were examined via western blotting. In some experiments cell lysis and recovery of ubiquitinated proteins was performed under denaturing conditions. For those experiments, Flag-TRIM69A (WT or E3 mut), HA-MST2 (WT or ubiquitination site mutant) and His-Myc-ubiquitin plasmids were co-transfected into cells. After 48 h, the transfected cells were treated with MG132 (20 μM) for 8 h. Cells were recovered by scraping and aliquots of the harvested cells (∼10%) were reserved for quantification of protein expression. The remaining cells were lysed with denaturing buffer A (6 M guanidine HCl, 0.1 M Na_2_HPO_4_/NaH_2_PO_4_ and 5 mM imidazole) and sonicated briefly. For each experimental condition, 2 mg of cell lysate was incubated with 50 μl of TALON Metal Affinity Resin (Takara, 635501) and rotated at 4°C overnight. The TALON beads were then sequentially washed once with buffer A, twice with buffer B (1.5 M guanidine HCl, 25 mM Na_2_HPO_4_/NaH_2_PO_4_, 20 mM Tris–Cl pH 6.8 and 10 mM imidazole) and three times with buffer T1 (25 mM Tris–Cl pH 6.8 and 15 mM imidazole). After the washes, beads were boiled in 100 μl of 2× Laemmli loading buffer containing 200 mM imidazole to release the ubiquitinated proteins. Beads were removed by centrifugation and released proteins were analyzed by SDS-PAGE and immunoblotting.

### Luciferase assay

Luciferase activities were measured by using the dual luciferase reporter assay (Promega) according to the manufacturer's protocol. pRL/TK-luciferase reporter plasmid was used as a second reporter. The data were obtained by analyzing triplicated samples. In general, 100 ng expression plasmid, 60 ng 8xGTIIC-luciferase (Addgene), and 3 ng pRL-TK (internal control) were co-transfected into H1299 cells plated in 24-well plates. 48 h later, cells were harvested and luciferase activities were measured by using the dual luciferase reporter assay (Promega, Madison, WI, USA) according to the manufacturer's protocol. All reporter assays were completed at least in triplicate, and the results were shown as average values ± standard deviations (SD) from one representative experiment.

### Clonogenic survival assays

For experiments in H1299 RFP-H2B, MDA-MB-231 TRIM69A KO and U2OS, cells were seeded at a density of 2000 cells/well in triplicate in six-well plates. Cells were transfected with siRNA for 24 h before seeding to the plates. Growth medium was replenished every 3 days. Colonies were stained with 0.05% crystal violet in 1× PBS containing 1% methanol and 1% formaldehyde. The ImageJ plugin ColonyArea was used to automatically quantify stained colonies.

### Mass spectrometry

Proteomics preparation after affinity purification of TRIM69 isoforms and MST2: Immunoprecipitated protein samples were subjected to on-bead trypsin digestion as previously described (37). Briefly, after the last wash buffer step during affinity purification, beads were resuspended in 50 μl of 50mM ammonium bicarbonate (pH 8). On-bead digestion was performed by adding 1μg trypsin and incubating overnight at 37°C while shaking. The next day, 0.5μg trypsin was added and incubated at 37°C for an additional 3h. Beads were recovered by centrifugation and supernatants transferred to fresh tubes. The beads were washed twice with 100μl LC–MS grade water, and washes were added to the original supernatants. Samples were acidified by adding formic acid to final concentration of 2%. Peptides were desalted using peptide desalting spin columns (Thermo Fisher), lyophilized and stored at –80°C until further analysis.

For phosphoproteomics sample preparation, cell lysates (400 μg; *n* = 3) were lysed in 8M urea, reduced with 5mM DTT for 45 min at 37°C and alkylated with 15mM iodoacetamide for 30 min in the dark at room temperature. Samples were digested with LysC (Wako, 1:50 w/w) for 2 h at 37°C, then diluted to 1M urea and digested with trypsin (Promega, 1:50 w/w) overnight at 37°C. The resulting peptide samples were acidified to 0.5% trifluoracetic acid, desalted using desalting spin columns (Thermo Fisher), and the eluates were dried via vacuum centrifugation. Peptide concentration was determined using Quantitative Colorimetric Peptide Assay (Thermo Fisher).

Samples were labeled with TMTpro (Thermo Fisher), for a total of two TMTpro 16plex sets. 125 μg of each sample was reconstituted with 50 mM HEPES pH 8.5, then individually labeled with 250 μg of TMTpro reagent for 1 h at room temperature. Prior to quenching, the labeling efficiency was evaluated by LC–MS/MS analysis of a pooled sample consisting of 1 ul of each sample. After confirming > 98% efficiency, samples were quenched with 50% hydroxylamine to a final concentration of 0.4%. Labeled peptide samples were combined 1:1, desalted using Thermo desalting spin column, and dried via vacuum centrifugation. The dried TMT-labeled samples (six TMT sets total) were fractionated using high pH reversed phase HPLC (38). Briefly, the samples were offline fractionated over a 90 min run, into 96 fractions by high pH reverse-phase HPLC (Agilent 1260) using an Agilent Zorbax 300 Extend-C18 column (3.5-μm, 4.6 × 250 mm) with mobile phase A containing 4.5 mM ammonium formate (pH 10) in 2% (vol/vol) LC–MS grade acetonitrile, and mobile phase B containing 4.5 mM ammonium formate (pH 10) in 90% (vol/vol) LC–MS grade acetonitrile. The 96 resulting fractions were then concatenated in a non-continuous manner into 24 fractions and 5% of each were aliquoted, dried down via vacuum centrifugation and stored at –80°C until further analysis. The remaining 95% of each fraction was further concatenated into 3 fractions and dried down via vacuum centrifugation. For each fraction, phosphopeptides were enriched with the High Select Fe-NTA kit (Thermo Fisher) per manufacturer's protocol. The Fe-NTA eluates were dried down via vacuum centrifugation and stored at –80°C until further analysis.

### LC/MS/MS and data analysis

For the affinity purification samples: The peptide samples were analyzed in duplicate by LC/MS/MS using an Easy nLC 1200 coupled to a QExactive HF mass spectrometer (Thermo Fisher). Samples were injected onto an Easy Spray PepMap C18 column (75 μm id × 25 cm, 2 μm particle size) (Thermo Fisher) and separated over a 2 h method. The gradient for separation consisted of 5–45% mobile phase B at a 250 nl/min flow rate, where mobile phase A was 0.1% formic acid in water and mobile phase B consisted of 0.1% formic acid in ACN. The QExactive HF was operated in data-dependent mode where the 15 most intense precursors were selected for subsequent fragmentation. Resolution for the precursor scan (*m*/*z* 300–1600) was set to 120 000, while MS/MS scans resolution was set to 15 000. The normalized collision energy was set to 27% for HCD. Peptide match was set to preferred, and precursors with unknown charge or a charge state of 1 and ≥7 were excluded.

For the global phosphoproteomics samples: Two sets of 24 fractions for the proteome analysis and two sets of 3 FeNTA-enriched fractions for the phosphoproteome analysis were analyzed by LC/MS/MS using an Easy nLC 1200 coupled to an Orbitrap Fusion Lumos Tribrid mass spectrometer (Thermo Fisher). Samples were injected onto an Easy Spray PepMap C18 column (75 μm id × 25 cm, 2 μm particle size) (Thermo Fisher) and separated over a 120 min method. The gradient for separation consisted of 5–42% mobile phase B at a 250 nl/min flow rate, where mobile phase A was 0.1% formic acid in water and mobile phase B consisted of 0.1% formic acid in 80% ACN.

For the proteome fractions, the Lumos was operated in SPS-MS3 (39), with a 3s cycle time. Resolution for the precursor scan (m/z 400–1500) was set to 120000 with a AGC target set to standard and a maximum injection time of 50 ms. MS2 scans consisted of CID normalized collision energy (NCE) 32; AGC target set to standard; maximum injection time of 50 ms; isolation window of 0.7 Da. Following MS2 acquisition, MS3 spectra were collected in SPS mode (10 scans per outcome); HCD set to 55; resolution set to 50 000; scan range set to 100–500; AGC target set to 200% with a 100 ms maximum inject time.

For the phosphoproteome fractions, the Lumos was operated in MS2 (40) with a 3s cycle time. Resolution for the precursor scan (*m*/*z* 400–1500) was set to 60 000 with a AGC target set to standard and a maximum injection time of 50 ms. For MS2 scans, HCD was set to 35; AGC target set to 200%; maximum injection time of 120 ms; isolation window of 0.7 Da; resolution set to 50 000.

Raw data files were processed using Proteome Discoverer version 2.4 (Thermo Fisher) and searched against the reviewed human database (containing 20 203 entries), appended with a common contaminants database, using Sequest. Enzyme specificity was set to trypsin and up to two missed cleavage sites were allowed. For the affinity purification samples, methionine oxidation, N-terminus acetylation, serine/threonine/tyrosine phosphorylation and lysine diglycine were set as variable modifications. For TMT proteome and phosphoproteome samples, cysteine carbamidomethylation and TMTpro were set as a fixed modification on peptide N-terminus and lysine; methionine oxidation was set as a variable modification. For phosphoproteome samples, serine/threonine/tyrosine phosphorylation were set as variable modifications. The Percolator node was used to calculated false discovery rates (FDR). A peptide FDR of 1% was used to filter all data. For PTMs, the ptmRS node was used to localize modification sites. For the affinity purification samples, the Minora node was used to extract peak areas and the ‘Precursor Ions Quantifier’ node was used for relative quantitation of peptides/proteins across samples. For the proteome and phosphoproteome samples, the ‘Reporter Ions Quantifier’ node was used to extract reporter ion abundances (intensities). Normalization and statistical analysis to calculate p-values and log2 fold changes were all performed in Proteome Discoverer.

### Study populations and datasets

The Cancer Genome Atlas (TCGA) is a large collaboration aimed at conducting standardized molecular profiling of over 30 cancer types and has been described in detail elsewhere (41). We downloaded clinical, RNA-Seq, and somatic alteration data for primary breast tumors in TCGA using NCI Genomic Data Commons (GDC, https://gdc.cancer.gov/). Our primary gene expression analyses were limited to TRIM69 and a curated list of 39 centrosome-associated genes (CA20 Genes: AURKA, CCNA2, CCND1, CCNE1, CDK1, CEP63, CEP152, E2F1, E2F2, LMO4, MDM2, MYCN, NDRG1, NEK2, PIN1, PLK1, PLK4, SASS6, STIL, TUBG1; Other Centrosome Genes: NUP205, MST2, MST1, DYNC1H1, NUP133, NUP107, NUP214, NUP93, NUP210, PLK1, TUBB, CENPF, KIF11, DCTN1, NEK2, CEP250, CROCC, PPP1CC, SAV1, STAT3, STMN1). Normalized gene expression values were log_2_ transformed and median centered for all downstream analyses. We also profiled tumors for PAM50 subtype (42), an RNA-based classifier of p53 (43), and DNA repair phenotypes

### HRD, DNA damage, CA20 scores and heatmap

We used a number of previously published genomic algorithms to validate associations between TRIM69 expression, DNA damage response, and centrosome function. Briefly, continuous HRD scores were derived from Knijnenburg *et al.* (44), calculated using 3 components of HRD/genome scarring scores: HRD-Loss of Heterozygosity (LOH) (45), large scale transitions (46), number of sub chromosomal regions with allelic imbalance extending to the telomere (NtAI) (47), and the implementation of a sum of the three (44). Scores were dichotomized at a cut point of 42 in accordance with previous recommendations (44). In addition, DNA Damage Repair (DDR) scores, representing estimates of cumulative DNA damage, and aneuploidy scores, reflecting arm-level deletions and gains measured via ABSOLUTE, were also extracted from Knijnenburg *et al.* (44). Centrosome amplification scores (CA20) were taken as the sum of log2-transformed, median centered RNA expression values for 20 centrosome-associated proteins, using a method from de Almeida et al (16)

To visualize associations centrosome function, clinical characteristics, and global indicators of DNA damage, we constructed an annotated heatmap of centrosome-associated protein expression. Log_2_ transformed, median-centered gene expression values of 39 centrosome-associated proteins (CA20 Genes: AURKA, CCNA2, CCND1, CCNE1, CDK1, CEP63, CEP152, E2F1, E2F2, LMO4, MDM2, MYCN, NDRG1, NEK2, PIN1, PLK1, PLK4, SASS6, STIL, TUBG1; Other Centrosome Genes: NUP205, MST2, MST1, DYNC1H1, NUP133, NUP107, NUP214, NUP93, NUP210, PLK1, TUBB, CENPF, KIF11, DCTN1, NEK2, CEP250, CROCC, PPP1CC, SAV1, STAT3, STMN1) for 1094 breast cancer patients with associated clinical data were clustered using centroid, hierarchical linkage and plotted using the ComplexHeatmap package in R. To assess whether TRIM69 expression was associated with DNA damage and centrosome function, we conducted Wilcoxon rank-sign tests comparing CA20, Aneuploidy, and HRD scores between samples dichotomized as TRIM69-high (expression in the top quartile) versus TRIM69-low (expression in the bottom three quartiles). All analyses were performed in R, version 4.2.1.

### Statistics and reproducibility

Statistical analysis was performed using Microsoft Excel and GraphPad Prism 6. Student's *t*-test was used to determine *P* values for all data involving comparisons between two groups. Results are expressed as the mean ± standard error of the mean (SEM) of two independent experiments. *P* values are indicated in the Figure legends. Microscopy images shown are representative of at least 10 fields from two independent experiments. Western blot images are representative of two independent experiments. All biological and biochemical experiments were performed with appropriate internal negative and/or positive controls as indicated.

## RESULTS

### TRIM69 expression is associated with Centrosome Amplification 20 (CA20) gene expression signature in basal breast cancers

**Figure 1.**
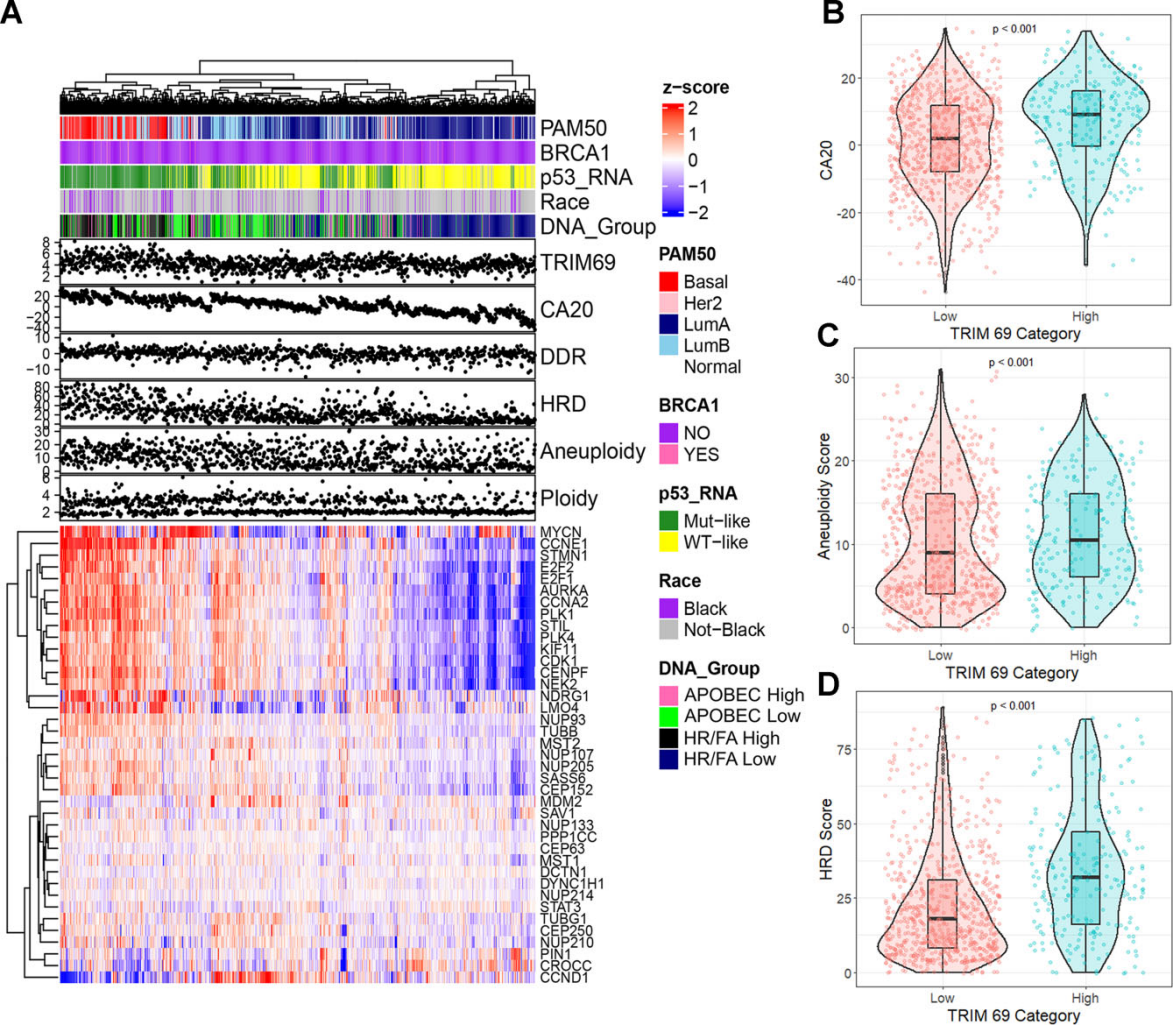
TRIM69A expression is associated with Centrosome Amplification 20 (CA20) gene expression signature, Homologous Recombination-Deficiency (HRD) and Aneuploidy in basal breast cancers. (**A**) Heatmap showing relative expression of CA20 signature genes in relation to *TRIM69A* mRNA expression and various other classifiers. Annotations for PAM50 subtype, *BRCA1* germline mutation status, *TP53* RNA subtype (43), self-reported race, and DNA Repair group are provided along with continuous CA20, DDR, and HRD signature scores. Heatmap clustering was performed by both samples (columns) and genes (rows) using centroid linkage. (**B**) Violin plot depicting distribution of CA20 scores by *TRIM69A* expression (low = bottom three quartiles, high = top quartile). P-value represents comparison of CA20 between *TRIM69A* high and low groups by Wilcoxon rank-sign test. (**C**) Violin plot of aneuploidy scores separated by *TRIM69A* expression group. P-value represents comparison of aneuploidy scores (44) in *TRIM69A* high versus low samples by Wilcoxon rank-sign test *t*. (**D**) Violin plot of HRD scores according to *TRIM69A* expression group. *P*-value represents comparison of HRD scores (44) in *TRIM69A* high versus low samples by Wilcoxon rank-sign test. This is a violin plot of samples separated as *TRIM69A* High or Low. HRD score is plotted on the y-axis. Wilcoxon *P*-value was performed.

We recently developed a predictive classifier of breast cancer subtypes based on mRNA expression profiling of DNA repair genes (48). In our analyses of TCGA data, we unexpectedly noticed that expression of the *TRIM69* mRNA in basal breast cancers was very strongly associated with gene signatures for centrosome amplification (CA20, Figure 1A, B), aneuploidy (Figure 1C), and Homologous Recombination Deficiency (HRD, Figure 1D). *TRIM69* encodes an E3 ubiquitin ligase of unknown function with possible roles in tolerance of mitotic stress (49). Interestingly, expression of the Cancer Testes Antigen (CTA) MAGE-A4, which encodes a pathological cancer-specific activating binding partner of TRIM69 (50) was also strongly associated with the basal subtype (48). Therefore, we sought to identify roles of TRIM69 and mechanistically define its putative role(s) in genome maintenance and mitotic processes.

### TRIM69A is dynamically associated with the centrosome during mitosis

**Figure 2.**
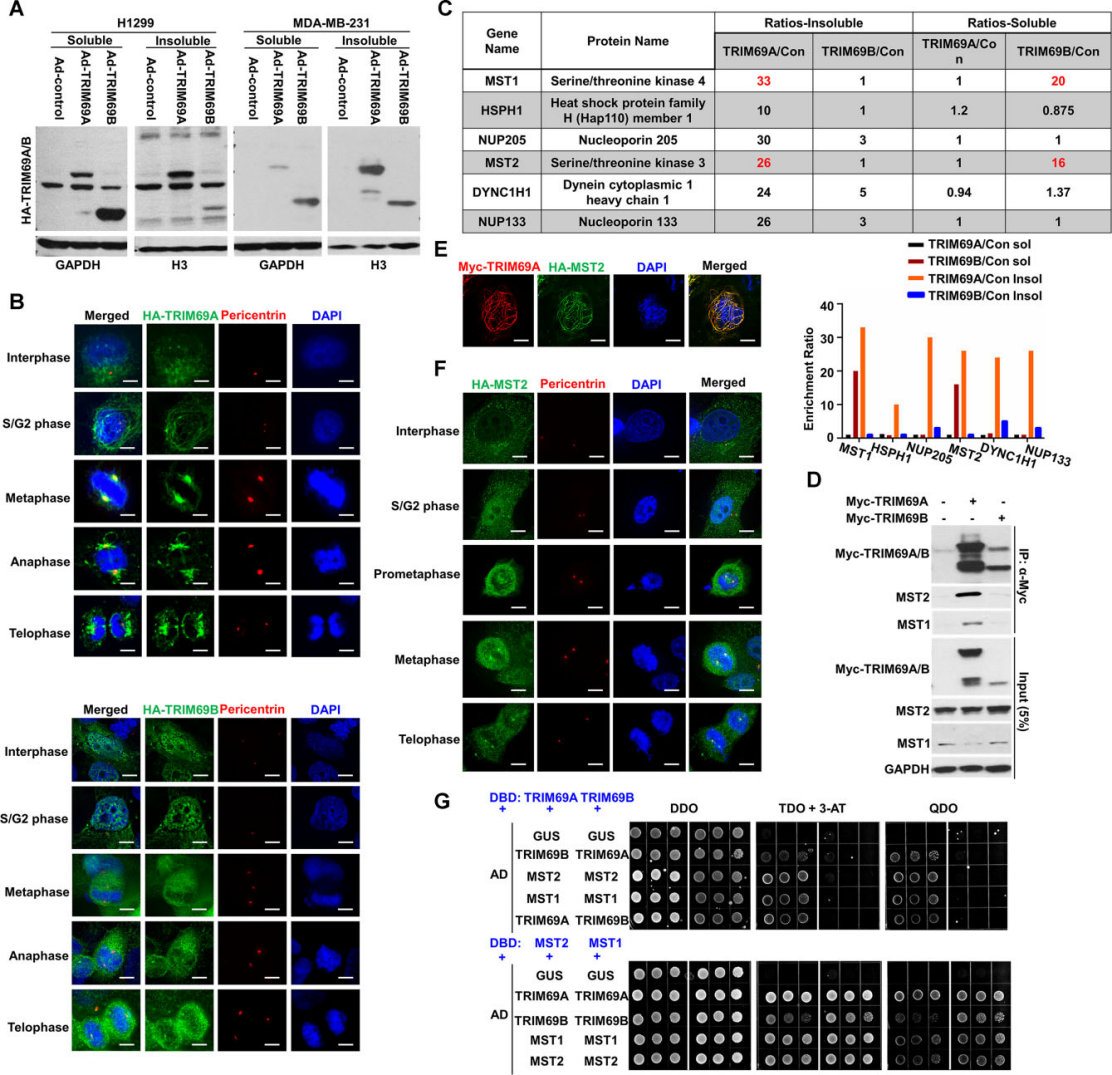
TRIM69A interacts with MST2 and MST1 mainly in a detergent-insoluble subcellular compartment. (**A**) Immunoblots showing relative distribution of TRIM69A and TRIM69B between detergent-soluble and detergent-insoluble cell fractions in H1299 and MDA-MB-231 cells. (**B**) Representative confocal microscopy images of HA-TRIM69A/B-expressing cells showing subcellular distribution of HA-TRIM69 (green) in relation to pericentrin (red) for each cell cycle stage (identified based on nuclear morphology). Scale = 5 μm. (**C**) H1299 cells transduced with viruses encoding HA-TRIM69A, HA-TRIM69B, or an ‘empty’ adenovirus vector for control. Cells were biochemically-fractionated to generate detergent-soluble and detergent-insoluble extracts. The detergent-insoluble extracts were dissociated using DNAse and sonication. HA-TRIM69A and HA-TRIM69B complexes were immunopurified from the extracts and analyzed by mass-spectrometry. The table lists the most abundant proteins that specifically co-purified from with HA-tagged TRIM69 variants. Max Ratios were selected with a stringent filter to avoid a ratio <2 for any sample. The bar chart shows fold-enrichment of various TRIM69-associated proteins in anti-HA-immunoprecipitates from HA-TRIM69A/B-expressing cells when compared with control (empty vector) cultures. (**D**) Immunoblot showing co-immunoprecipitation of MYC-TRIM69A with HA-MST2. (**E**) Confocal microscopy images of a representative H1299 cell showing co-localization of MYC-TRIM69A (red) with HA-MST2 (green). (**F**) Confocal microscopy images of representative H1299 cells at different cell cycle stages showing subcellular distribution of HA-MST2 (green) in relation to pericentrin (red). Scale = 5 μm. (**G**) Yeast 2-hybrid assays showing extent to which TRIM69A or TRIM69B interact with MST2 and MST1.

To define mechanisms by which TRIM69 regulates centrosomes and mitosis we first determined the subcellular localization of the TRIM69 protein. The TRIM69 transcript encodes two variant proteins, TRIM69A and a smaller truncated species termed TRIM69B which lacks the N-terminal RING domain (49,51) ( Supplementary Figure S1A). We ectopically expressed HA-tagged versions of TRIM69A and TRIM69B in MDA-MB-231 breast cancer and in H1299 lung adenocarcinoma cells using adenoviral vectors, then determined subcellular distribution of the two TRIM69 variants using biochemical fractionation and immunoblotting. As shown in Figure 2A, TRIM69A primarily localized to the detergent-insoluble perinuclear cytoskeleton (CSK) fraction while TRIM69B was largely detergent-soluble in both MDA-MB-231 and H1299 cells.

To investigate connections between TRIM69 and centrosomes we determined more precisely the subcellular distribution of TRIM69 in cells transitioning from S-phase to mitosis. Interestingly, in S-phase, TRIM69A localized to brightly-stained fiber arrays which encircle the nucleus (Figure 2B upper panel). On entering mitosis TRIM69A became highly concentrated at the centrosomes and was also detectable in the astral and kinetochore microtubules. In anaphase and telophase, TRIM69A co-localization with the centrosomes was progressively reduced, yet TRIM69A remained associated with the microtubule networks. During telophase, TRIM69A also formed a ring around the periphery of the daughter nuclei, likely corresponding to the nascent microtubule fibers encompassing new nucleus. Unlike TRIM69A, TRIM69B was distributed broadly throughout the cell and did not localize specifically with the centrosome (Figure 2B, lower panel). The dynamic association of TRIM69A with the centrosome is fully consistent with a fundamental role for this E3 ligase in regulating its dynamics.

### MST2 and MST1 are novel interaction partners of TRIM69A

To identify potential effectors of TRIM69 in regulating centrosome dynamics we performed mass-spectrometry analysis of immunopurified TRIM69 complexes and defined the TRIM69 protein-interaction network. As shown in Figure 2C, we identified the Serine/Threonine protein Kinases MST2 and MST1 as the most abundant components of the TRIM69 complex. Other TRIM69-associated proteins we identified included Nuclear Pore proteins NUP205 and NUP133, as well as Dynein Cytoplasmic 1 Heavy Chain 1 (DYNC1H1). The MST2/1 kinases mediate the Hippo pathway which controls cell proliferation, mitosis and cell polarity (52). Moreover, the Hippo pathway is triggered in part by extra centrosomes (53). Therefore, we initially focused on MST2/1 as potential components of TRIM69 signaling in a pathway regulating centrosome dynamics.

We performed independent co-immunoprecipitation experiments to validate the mass spectrometry experiments and demonstrate that MST2/1 are members of the TRIM69 complex (Figure 2D). Using immunofluorescence microscopy we observed co-localization of MST2 and TRIM69 (Figure 2E). Similar to TRIM69A, HA-MST2 co-localized with centrosomes during mitosis (Figure 2F), consistent with a potential role for TRIM69 and MST2 in regulating centrosomes. To determine the mechanism of interaction between TRIM69 and MST2/1, we performed yeast two-hybrid (Y2H) assays. As shown in Figure 2G, our Y2H assays detected strong reciprocal interactions between TRIM69A and MST2/1 (Figure 2G, upper and lower panels), indicating that TRIM69A interacts directly with MST2/1. By comparison, TRIM69B interactions with MST2/1 were weak and barely detectable. We conclude that MST2 and MST1 are novel TRIM69A-binding partners.

**Figure 3.**
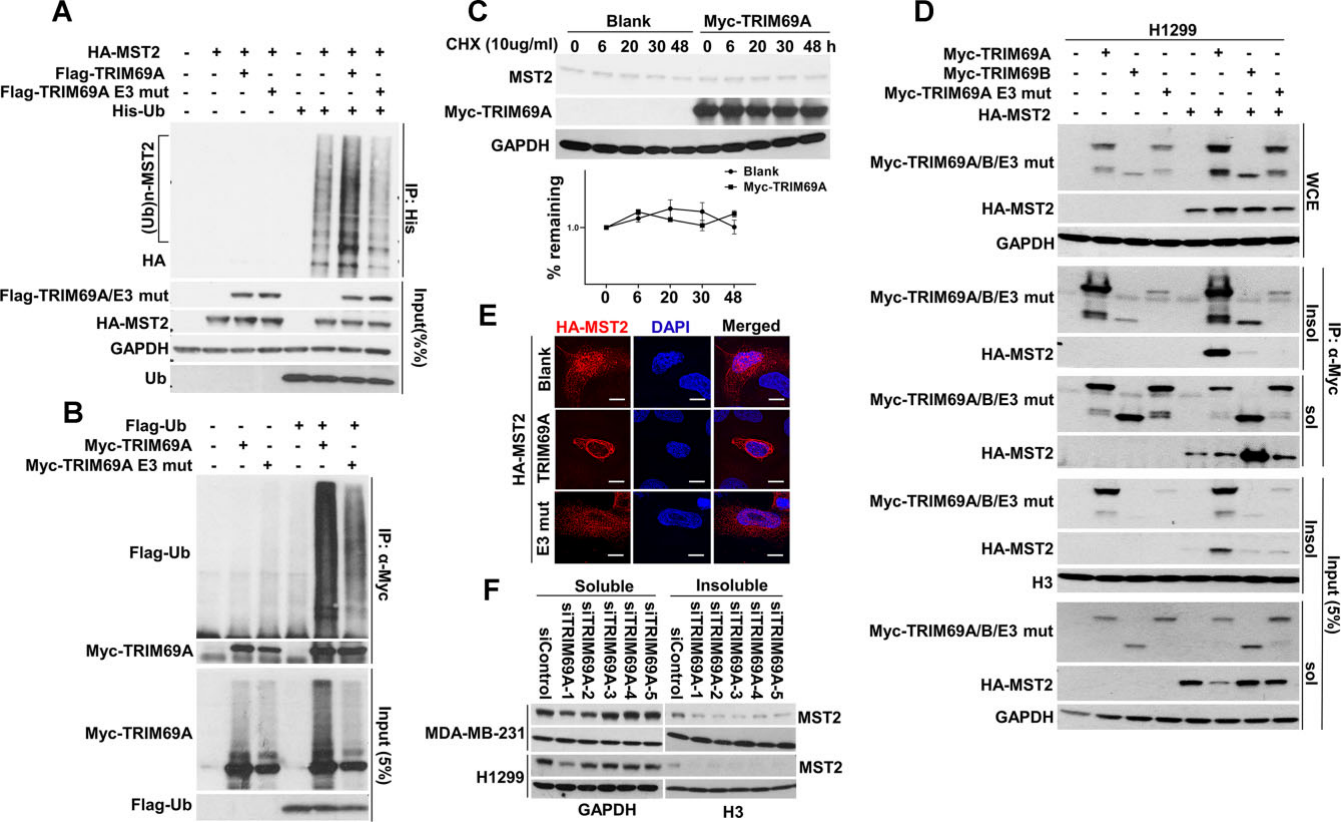
TRIM69A regulates MST2 ubiquitination and subcellular distribution. (**A**) TRIM69 (WT), but not catalytically-inactive TRIM69A (E3 mut) promotes conjugation of Ub to MST2. (**B**) TRIM69 WT but not catalytically-inactive TRIM69A (E3 mut) undergoes autoubiquitination. (**C**) MYC-TRIM69A does not affect MST2 stability in cycloheximide (CHX, 10 μg/ml)-treated 239T cells. The half-life of MST2 was determined based on results from two different independent experiments. Relative levels of MST2 as detected by immunoblotting were quantified using ImageJ software and normalized to GAPDH levels. (**D**) Wild-type MYC-TRIM69A (but not MYC-TRIM69A E3 mut or MYC-TRIM69B) redistributes MST2 to the detergent insoluble compartment in H1299 cells. (**E**) Confocal microscopy images of representative H1299 cells showing TRIM69A-induced subcellular redistribution of HA-MST2. Scale bar represents 5 μm. (**F**) Immunoblot showing that multiple independent TRIM69-directed siRNAs reduce the amount of endogenous MST2 associated with the detergent-insoluble compartment in H1299 and MDA-MB-231 cells.

Next, we asked whether TRIM69A promotes MST2/1 ubiquitination. We analyzed levels of MST2 ubiquitination in cells over-expressing wild-type TRIM69A or a catalytically-inactive TRIM69A mutant harboring C > A substitutions in amino acids 61 and 64 of the RING domain (54). As shown in Figures 3A-B, TRIM69A was auto-ubiquitinated and also stimulated MST2 ubiquitination. However, auto-ubiquitination activity was reduced by ∼60% for the catalytically-inactive TRIM69A mutant when compared with WT TRIM69A. MST2-induced ubiquitination activity was also reduced (by ∼70%) in cells expressing catalytically-inactive TRIM69A when compared with cells expressing WT TRIM69A (Figure 3A, B). We conclude that MST2 is likely to be a TRIM69 substrate. However, we have not observed any effect of TRIM69 on MST2 levels or protein stability (Figure 3*C*). Therefore, TRIM69-dependent MST2 ubiquitination is most likely unrelated to proteasomal degradation.

To determine the type of TRIM69-induced polyubiquitin chain linkage assembled on MST2, we co-expressed MST2 with a panel of ubiquitin mutants, for which each has only one of the seven possible lysines available for polymer chain assembly (55). As shown in Supplementary Figure S1B, TRIM69A promoted both K6- and K29- , but not K48-linked ubiquitination of MST2. This result suggests that TRIM69A-mediated ubiquitination does not promote proteasome-mediated degradation of MST2.

Our proteomic analysis of the MST2 complex from cells ectopically expressing TRIM69A revealed a di-Gly ubiquitin remnant at K279 of MST2. Therefore, we tested a K279 > A MST2 mutant for TRIM69A-dependent ubiquitination. Our results showed that this bona-fide ubiquitination site at K279 is not absolutely required for TRIM69A-dependent ubiquitination of MST2. The caveat of this experiment is that while many E3 ligases have a preferred ubiquitination site on their substrate proteins, mutating that preferred site will often result in ubiquitin conjugation at alternative lysine residues. Often, it is necessary to remove many, or even all lysine residues on a target protein in order to abrogate ubiquitination. Therefore, based on our results with the K279A mutant we can not exclude the possibility that K279 is a TRIM69A target site. There are 6 other lysine residues in the vicinity (∼40 AAs) of K279 and it is possible that those residues are targeted for ubiquitination when K279 is unavailable.

We noticed that ectopically-expressed TRIM69A promoted redistribution of HA-MST2 to a chromatin- and cytoskeleton-enriched detergent-insoluble CSK fraction (see 'Input' panels of Figure 3D). We also detected robust association of TRIM69A and MST2 in the detergent-insoluble CSK fraction. Interestingly, the RING finger TRIM69A mutant (TRIM69A E3 mut) failed to redistribute MST2 to the CSK-insoluble fraction. The truncated TRIM69B variant (which lacks the RING domain) was also unable to redistribute MST2 to the CSK compartment (Figure 3D). In immunofluorescence experiments, HA-MST2 was distributed broadly in the cell in the absence of TRIM69A (Figure 3E). However, HA-MST2 was redistributed to filamentous structures encircling the nucleus when co-expressed with WT TRIM69A (but not the TRIM69A RING finger mutant, see Figure 3E). Consistent with a role for TRIM69 in regulating MST2 subcellular localization, depleting endogenous TRIM69 using siRNA decreased the amount of CSK-associated MST2 (Figure 3F). Taken together, the results of Figure 2 identify MST2 as a novel TRIM69A binding partner and substrate. Moreover, the RING domain of TRIM69A is critical for both associating with and regulating the subcellular localization of MST2.

### TRIM69/STK interactions do not affect the Hippo pathway

MST2 and MST1 are core protein kinases of the Hippo pathway, a conserved signal transduction cascade that controls transcriptional programs involved in diverse processes including cell proliferation, survival (56), and the centrosome pathway (57). Therefore, we tested a role for TRIM69-STK signaling in regulating the Hippo signaling cascade and its transcriptional endpoints. As shown in Supplementary Figure S2 (panels A and B), neither depletion nor overexpression of TRIM69A affected levels of phosphoproteins (such as p-MOB1, P-YAP) that critically regulate the Hippo pathway. Knockdown efficiency of TRIM69A in these experiments is shown in Supplementary Figure S2C. Similarly, TRIM69 ablation or overexpression did not affect Hippo pathway transcriptional endpoints. Staurosporine-induced repression of Hippo-responsive genes such as CYR61, CTGF, and ANKRD1 (Supplementary Figure S2D) was unaffected by TRIM69 status (Supplementary Figure S2E). As expected, ectopically-expressed MST2 did repress luciferase reporter activity driven by a Hippo pathway-responsive 8xGTIIC promoter (Supplementary Figure S2F). However, Hippo pathway reporter gene activity was unaffected by TRIM69 (Supplementary Figure S2G). Therefore, TRIM69-MST2/1 interaction does not regulate centrosome behaviour and mitotic progression via the Hippo pathway.

### TRIM69A stimulates phosphorylation of MST2 by PLK1 and promotes centrosome disjunction

**Figure 4.**
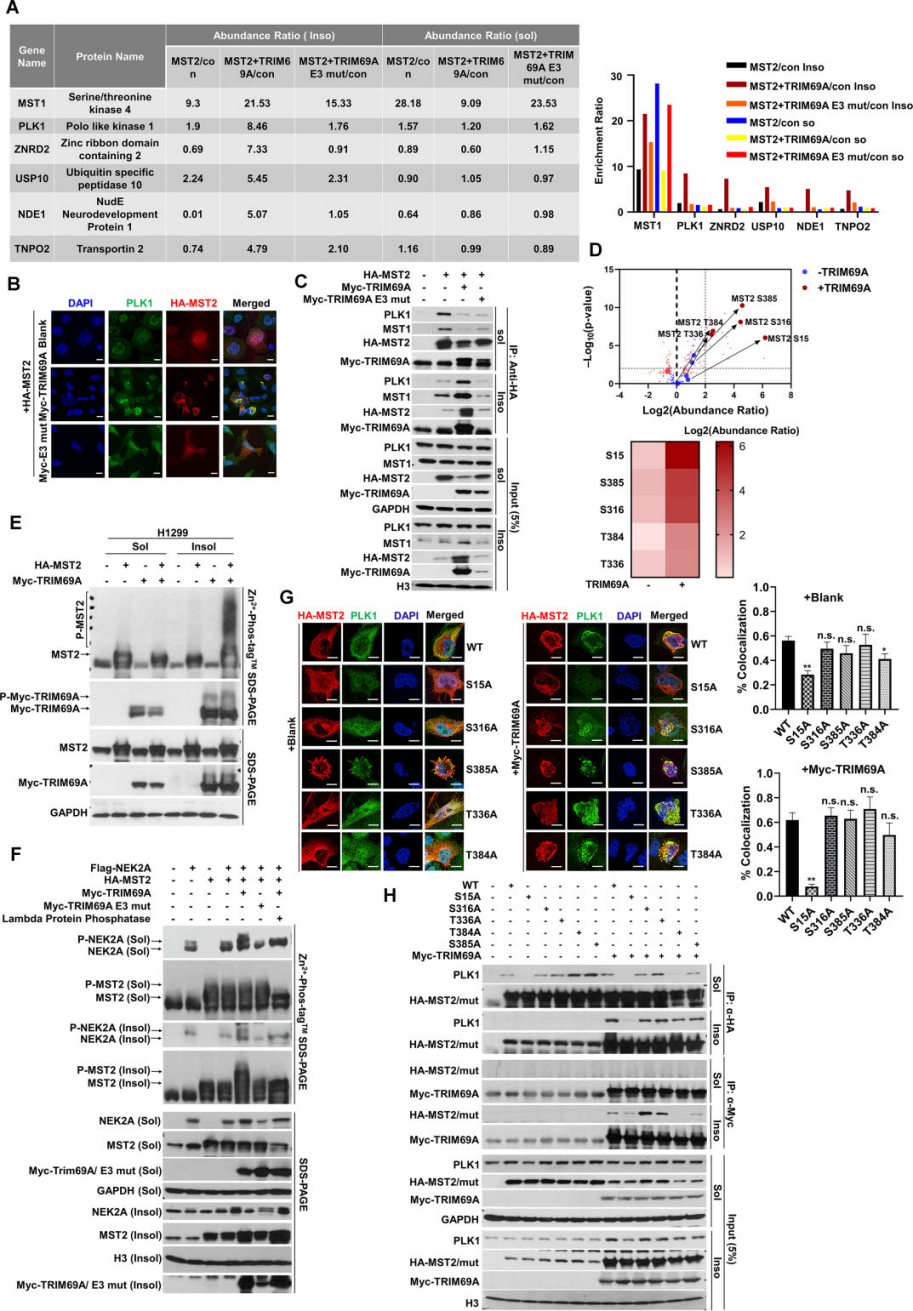
TRIM69A promotes MST2-PLK1 interaction in the detergent-insoluble compartment. (**A**) Effect of TRIM69 on the protein-interaction network of MST2 in detergent-soluble and detergent-insoluble compartments. 1% FDR was used to filter all peptides/proteins; only proteins with >1 peptides are reported. (**B**) TRIM69A promotes co-localization of MST2 (red) and PLK1 (green) in H1299 cells. Scale bar represents 10 μm. (**C**) TRIM69A promotes interaction between MST2 and PLK1 in the detergent-insoluble compartment. (**D**) Phosphoproteome profiling analysis showing TRIM69-inducible MST2 phosphorylation sites in the detergent-insoluble compartment. 1% FDR was used to filter all peptides/proteins; only proteins with >1 peptides are reported. (**E**) Immunoblots showing phosphorylation of MST2 by TRIM69A in CSK insoluble fraction using SDS-PAGE and Phos-tag phosphate-affinity gel electrophoresis. (**F**) Immunoblots showing TRIM69A promoted phosphorylation of NEK2A by MST2 in CSK insoluble fraction using SDS-PAGE and Phos-tag phosphate-affinity gel electrophoresis. (**G**) Effect of MST2 phosphorylation site mutations on co-localization of MST2 and PLK1. Co-localization was quantified using the ImageJ plug-in JAcoP. Each column represents the mean ± standard error of the mean (SEM) from two independent experiments, *n* = 20 cells for each condition, ∗∗*P* < 0.01. Scale bar represents 5 μm. (**H**) Effect of MST2 phosphorylation site mutations on MST2 interactions with TRIM69A and PLK1.

To elucidate pathways regulated by TRIM69- MST2 signaling, we defined the MST2 interactome in the presence and absence of co-expressed TRIM69A. We identified several high-confidence TRIM69A-inducible MST2 interactors in the detergent-insoluble CSK fraction including: MST1 (a known MST2 heterodimerization partner); zinc ribbon domain containing 2 (a centromeric protein); USP10 (a de-ubiquitinating enzyme); NDE1 (a centrosomal protein which regulates dynein function and microtubule organization); and TNPO2 (which mediates docking of the importin/substrate complex to the nuclear pore complex); and PLK1 (a proximal kinase involved in centrosome disjunction). Importantly, MST2-binding of these proteins was induced specifically by WT TRIM69A and not by the catalytically-inactive TRIM69A mutant (Figure 4A).

In addition to its canonical role in the Hippo pathway, MST2 mediates PLK1-induced centrosome separation by promoting recruitment of NEK2A to the linked centrioles. NEK2A subsequently phosphorylates C-NAP1 and rootletin to stimulate centrosome disjunction (7). Therefore, we tested a role for TRIM69 in regulating PLK1-MST2 signalling and the centrosome cycle.

As shown in Figure 4C, WT TRIM69A (but not the E3 ligase mutant) promoted complex formation between MST2 and PLK1 in the detergent-insoluble CSK compartment. Consistent with our co-IP results, we also detected TRIM69A-inducible co-localization of HA-MST2 with PLK1 (Figure 4B). PLK1-mediated phosphorylation of MST2 at S15, S18 and S316 is an important event in centrosome disjunction (7). Analysis of MST2 phospho-peptides from the LC–MS/MS experiment revealed increased phosphorylation of S15, S316, T336, T384, S385 and several other residues in the detergent-insoluble fraction of TRIM69A-overexpressing cells (Figure 4D). We further validated TRIM69-dependent phosphorylation of MST2 using Phos-tag phosphate affinity gel electrophoresis (58). As shown in Figure 4E, Phos-tag gel electrophoresis revealed a TRIM69A-inducible phosphorylated MST2 species (labelled ‘P-MST2’ in Figure 4E). TRIM69A-induced phosphorylation of MST2 was also detectable using a phospho-specific antibody against pS316 (Supplementary Figure S3C). To determine whether there were preferred TRIM69-inducible MST2 phosphorylation sites, we constructed a series of MST2 mutants containing individual or combinatorial S15A/S316A/T336A/T384A/S385A substitutions. TRIM69A-induced MST2 phosphorylation was not significantly affected for any of the MST2 mutants harbouring individual alanine substitutions in S15, S316, T336, T384 or S385. However, mutating all five residues completely abolished TRIM69A-induced MST2 phosphorylation (Supplementary Figure S3A,B). We conclude that TRIM69A promotes multi-site phosphorylation of MST2. During the centrosome cycle, MST2 phosphorylates the downstream protein kinase NEK2A to promote centrosome disjunction (7). Our Phos-tag gel electrophoresis experiments also revealed that TRIM69A promotes NEK2A phosphorylation by MST2 (Figure 4F), further consistent with a role for TRIM69 in activating the MST2-NEK2A signaling cascade.

Next we performed co-immunoprecipitations to determine how defective MST2 phosphorylation impacts association with its binding partners. Remarkably, the TRIM69A-induced association of HA-MST2 with PLK1 was abrogated in the MST2 S15 > A mutant, but not the other phosphorylation-resistant MST2 variants (Figure 4H). We used IF microscopy to quantify co-localization of PLK with WT and phosphorylation site mutant forms of MST2. Figure 4G shows that co-localization of MST2 S15 > A with PLK1 was specifically reduced when compared with MST2 WT. These results are suggestive of a ternary complex involving TRIM69, PLK, and MST2 that is both positively and negatively regulated by different MST2 phosphorylation events.

**Figure 5.**
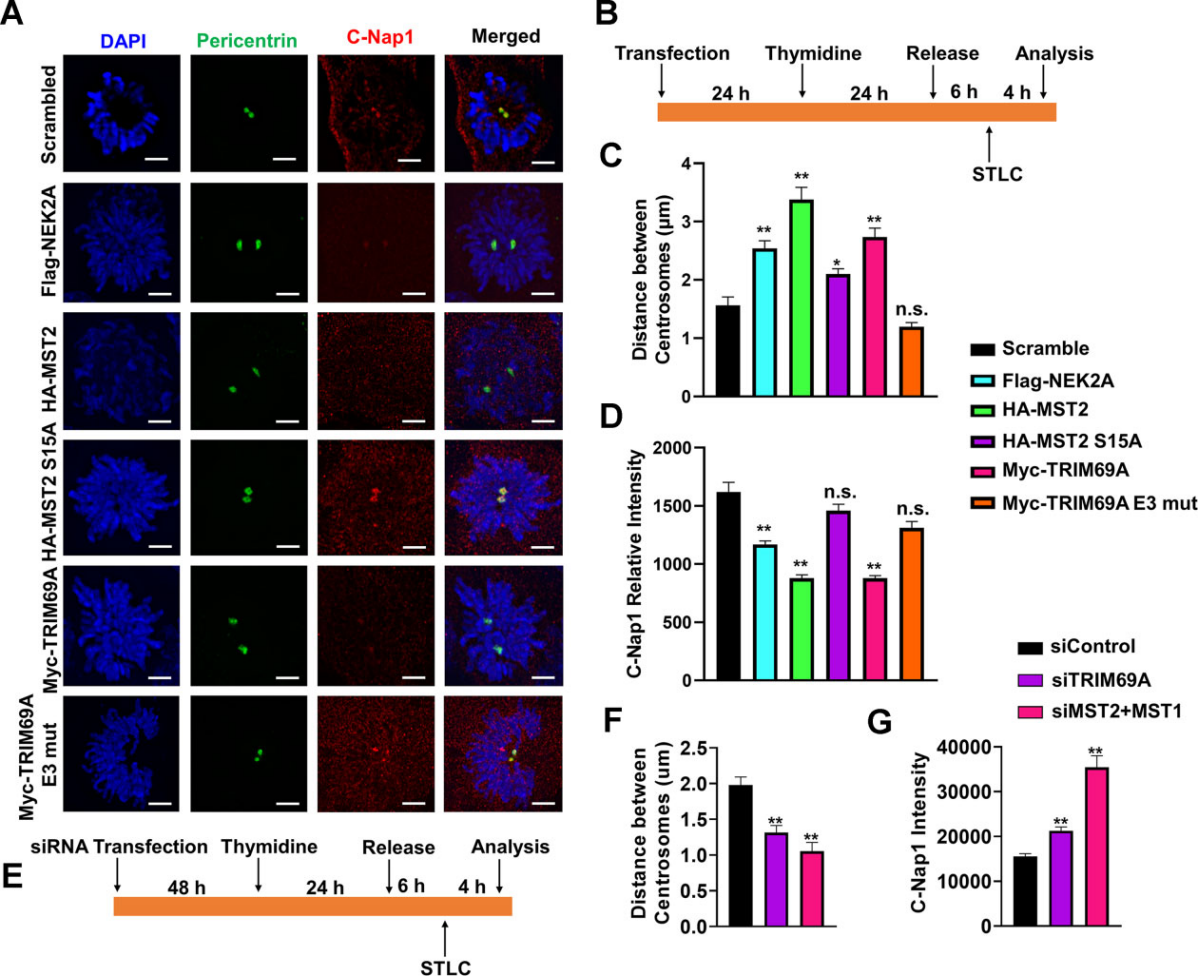
TRIM69 and MST2 promote centrosome separation. (**A**–**D**) U2OS cells were transfected with the indicated plasmids for 24 h before a single thymidine block/release and treatment with 5 mM Eg5 inhibitor (STLC) to trap cells in prometaphase. (A-B) Representative confocal microscopy images showing simultaneous immunostaining for pericentrin (green) and C-Nap1 (red) in U2OS cells harboring ectopically-expressed NEK2A, MST2, or TRIM69A. Scale = 5 μm. (C) Effect of ectopically-expressed NEK2A, MST2, or TRIM69A on inter-centrosome distance. Results are from two independent experiments. *n* > 20 cells were analyzed for each condition. Data are mean ± standard error of the mean (SEM) (∗∗*P* < 0.0001). (D) Effect of ectopically-expressed NEK2A, MST2 or TRIM69A on intensity of C-NAP1 signals at centrosomes. Results are from two independent experiments. *n* > 20 cells were analyzed for each condition. Data are mean ± standard error of the mean (SEM) (∗∗*P* < 0.0001). (**E, F**) Effect of siTRIM69 and siMST2/1 on inter-centrosome distance. Results are from two independent experiments; *n* > 20 cells were analyzed for each condition. Data are mean ± standard error of the mean (SEM) (∗∗*P* < 0.0001). (**G**) U2OS cells were transfected with indicated siRNAs for 48 h before a single thymidine block/release and treatment with 5 uM Eg5 inhibitor (STLC) to trap cells in prometaphase. The bar chart shows the effect of siTRIM69 and siMST2/1 on intensity of C-NAP1 signals at centrosomes. Results are from two independent experiments. *n* > 20 cells were analyzed for each condition. Data are mean ± standard error of the mean (SEM). ∗∗*P* < 0.0001.

To directly test the role of TRIM69A in regulating centrosome separation we determined the effect of TRIM69A overexpression or ablation on inter-centriolar distance. As reported previously (7), MST2 or NEK2A overexpression reduced the centrosomal staining of C-NAP1 (Figure 5A–D) and reduced the number of cells with low inter-centriolar distance (Figure 5C). Interestingly, TRIM69A overexpression fully phenocopied the effects of overexpressed MST2 or NEK2A (our positive controls for stimulation of centrosome disjunction) (Figure 5A–D) Conversely, TRIM69 ablation led to an increase in the number of unseparated centrioles and increased C-NAP1 intensity relative to control cells (Figure 5E–G). What's more, MST2 S15A mutant which abolished the interaction with PLK1 partially inactivates MST2 in promoting centrosome disjunction (Figure 5A–D). Taken together, the results of Figure 4 and Figure 5 suggest that TRIM69A promotes PLK1-mediated phosphorylation of MST2 to facilitate the disjunction phase of the centrosome cycle.

### TRIM69A-deficiency induces centrosome scattering and proliferation defects

**Figure 6.**
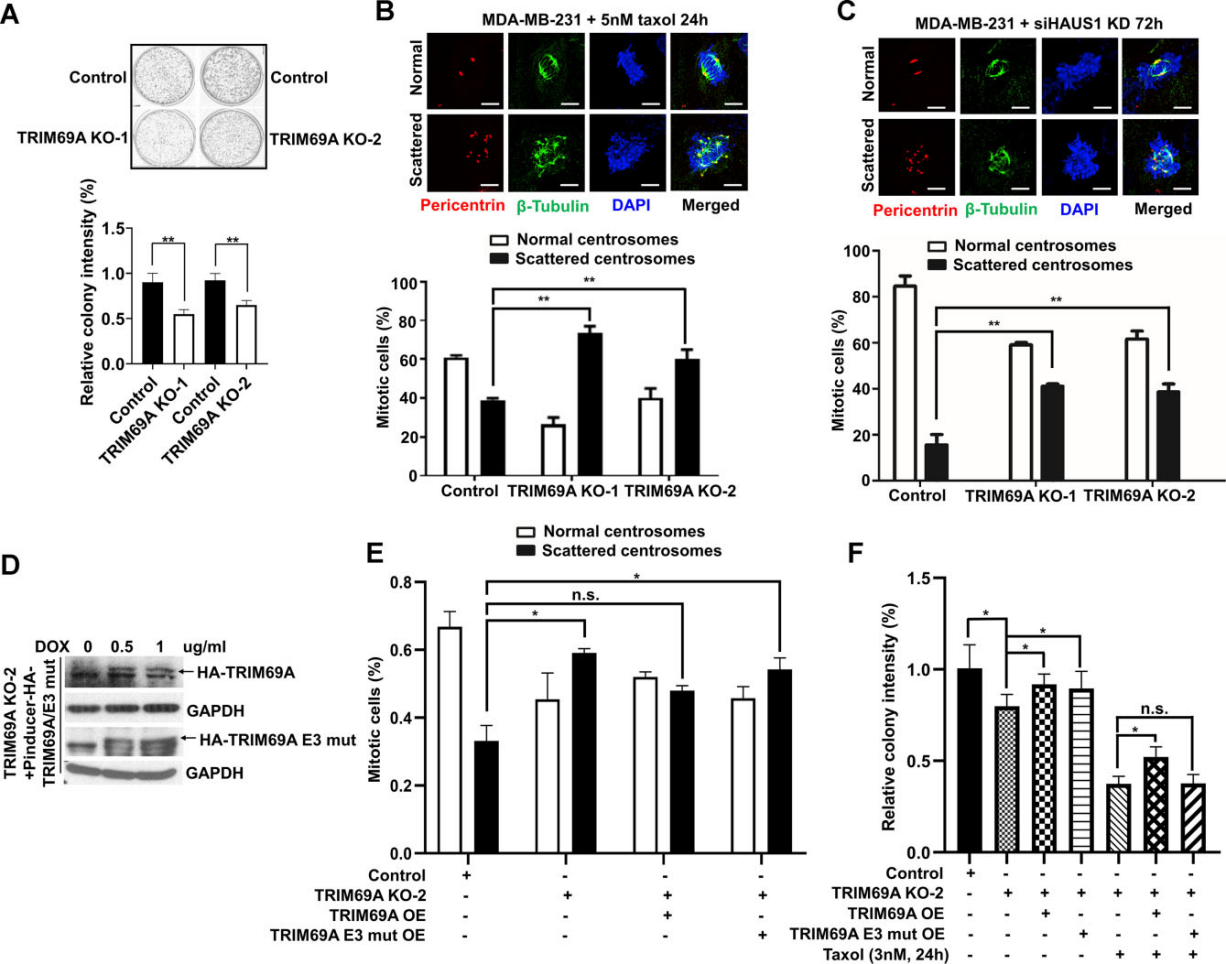
TRIM69A promotes triple-negative breast cancer (TNBC) cell proliferation and allows breast cancer cells to tolerate mitotic stresses due to centrosome amplification. (**A**) Clonogenic survival assays using parental MDA-MB-231 (*TRIM69^+/+^*) cells or a *TRIM69^−/−^* derivative cell line. Quantitative analysis of colony formation is presented in a bar chart (lower panel). Clonogenic survival of *TRIM69^−/−^* cells was normalized to colony survival of WT cells. Data points represent the mean of triplicate determinations ± SEM. **P ≤ 0.001. (**B**) Immunofluorescence images of pericentrin (red) and β-tubulin (green) staining in taxol-treated MDA-MB-231 cells showing representative normal and clustered centrosomes. Scale = 5 μm. The bar chart shows quantification of normal vs. scattered centrosomes in *TRIM69^+/+^* and *TRIM69^−/−^* derivative cells. Each column represents the mean ± range from two independent experiments, *n* = 100 cells for each condition. (**C**) Immunofluorescence images of pericentrin (red) and β-tubulin (green) staining in HAUS1 siRNA-treated MDA-MB-231 cells showing representative normal and clustered centrosomes. Scale = 5 μm. The bar chart shows quantification of cells with normal or scattered centrosomes. Bars represent the mean ± range from two independent experiments, *n* = 100 cells for each condition. (**D**) Immunoblots showing doxycycline-inducible expression of TRIM69A WT or TRIM69A E3 mut. (**E**) The bar chart shows quantification of normal versus scattered centrosomes in *TRIM69^+/+^* and *TRIM69^−/−^* cells after reconstituting TRIM69A WT or TRIM69A E3 mut. Each column represents the mean ± range from two independent experiments, *n* = 50 cells for each condition. ∗*P* < 0.05. (**F**) The bar chart shows quantification of colony formation in *TRIM69^+/+^* and *TRIM69^−/−^* cells after inducing TRIM69A WT or TRIM69A E3 mut expression with or without taxol treatment. Data points represent the mean of triplicate determinations ± SEM. *P < 0.05.

Given the high expression of TRIM69 in CA20-high cancers (Figure 1), we considered the possibility that TRIM69 and MST2 may also have roles in promoting centrosome clustering and averting mitotic defects that can arise from multipolar spindles (10,20,59). For these experiments we initially chose to work with the MDA-MB-231 breast cancer cell line which harbors amplified centrosomes (60). We used gene editing to generate *TRIM69^−/−^* clonal derivatives of MDA-MB-231 cells (Supplementary Figure S4). When compared with the parental *TRIM69^+/+^* MDA-MB-231 cells, *TRIM69^−/−^* cells showed decreased clonogenic survival (Figure 6A). We quantified centrosomes in *TRIM69^+/+^* and *TRIM69^−/−^* cells that were treated with 5 nM taxol, a first-line chemotherapeutic agent which stabilizes microtubules and causes spindle-multipolarity (22). As shown in Figure 6B, two independent clones of *TRIM69^−/−^* cells showed increases in spindle multipolarity and centrosome scattering when compared with parental MDA-MB-231 cells. In a complementary approach to determine the effect of TRIM69-deficiency on centrosome dynamics, we ablated HAUS1, a factor required for centrosome integrity (61) in *TRIM69^+/+^* and *TRIM69^−/−^* cells and quantified scattered centrosomes. As expected, HAUS1-depletion led to an ∼2-fold increase in centrosome scattering in two independent *TRIM69^−/−^* clones when compared with parental TRIM69 wild-type MDA-MB-231 cells (Figure 6C). We used Doxycycline-regulated promotors to conditionally reconstitute TRIM69A expression in *TRIM69^−/−^* cells (Figure 6D). Expression of WT TRIM69A (but not of catalytically-inactive TRIM69A E3 mut) rescued the centrosome scattering in *TRIM69^−/−^* cells (Figure 6E). Ectopically-expressed WT TRIM69A also promoted taxol-resistant cell proliferation (Figure 6F). The results of Figure 6 suggest that TRIM69 allows cancer cells to tolerate mitotic stresses by promoting centrosome clustering. High-level expression of TRIM69 in basal breast cancers (Figure 1) may represent an adaptive mechanism for tolerance of spindle multipolarity.

### TRIM69 regulates centrosome dynamics and mitotic progression

TRIM69 ablation in H1299 lung cancer cells (which harbor supernumerary centrosomes) also led to reduced clonogenic survival (Supplementary Figure S5A). Similar to MDA-MB-231 cells, treatment of H1299 cells with HAUS1 siRNA led to spindle multipolarity, which was partially rescued by ectopic over-expression of MYC-TRIM69A (Supplementary Figure S5B). Human cells with excessive centrosomes and multipolar spindles experience prolonged mitosis (62). To determine whether TRIM69/MST2 facilitate mitosis, we used time-lapse microscopy to study cell cycle progression of control (siCon), TRIM69-depleted, or MST2/MST1-depleted H1299 cells in the presence of paclitaxel. As shown in Supplementary Figure S5C–E, siRNA-mediated ablation of MST2/MST1 or TRIM69A led to prolonged mitosis. The mean times between nuclear envelope breakdown (NEB) and anaphase in control, TRIM69A-, and MST2/1-ablated cells were 53, 86 and 75 min, respectively (Supplementary Figure S5D, E). TRIM69A or MST2/1-depletion also led to increases in paclitaxel-dependent micronucleation when compared to the control cultures (Supplementary Figure S5F). Finally, depleting TRIM69 or MST2 and MST1 led to enhanced centrosome scattering and multipolar spindles in the presence of paclitaxel, fully recapitulating the effects of TRIM69A ablation (Supplementary Figure S5G). Knockdown efficiency of TRIM69A and MST2/1 is shown in Supplementary Figure S5H.

**Figure 7.**
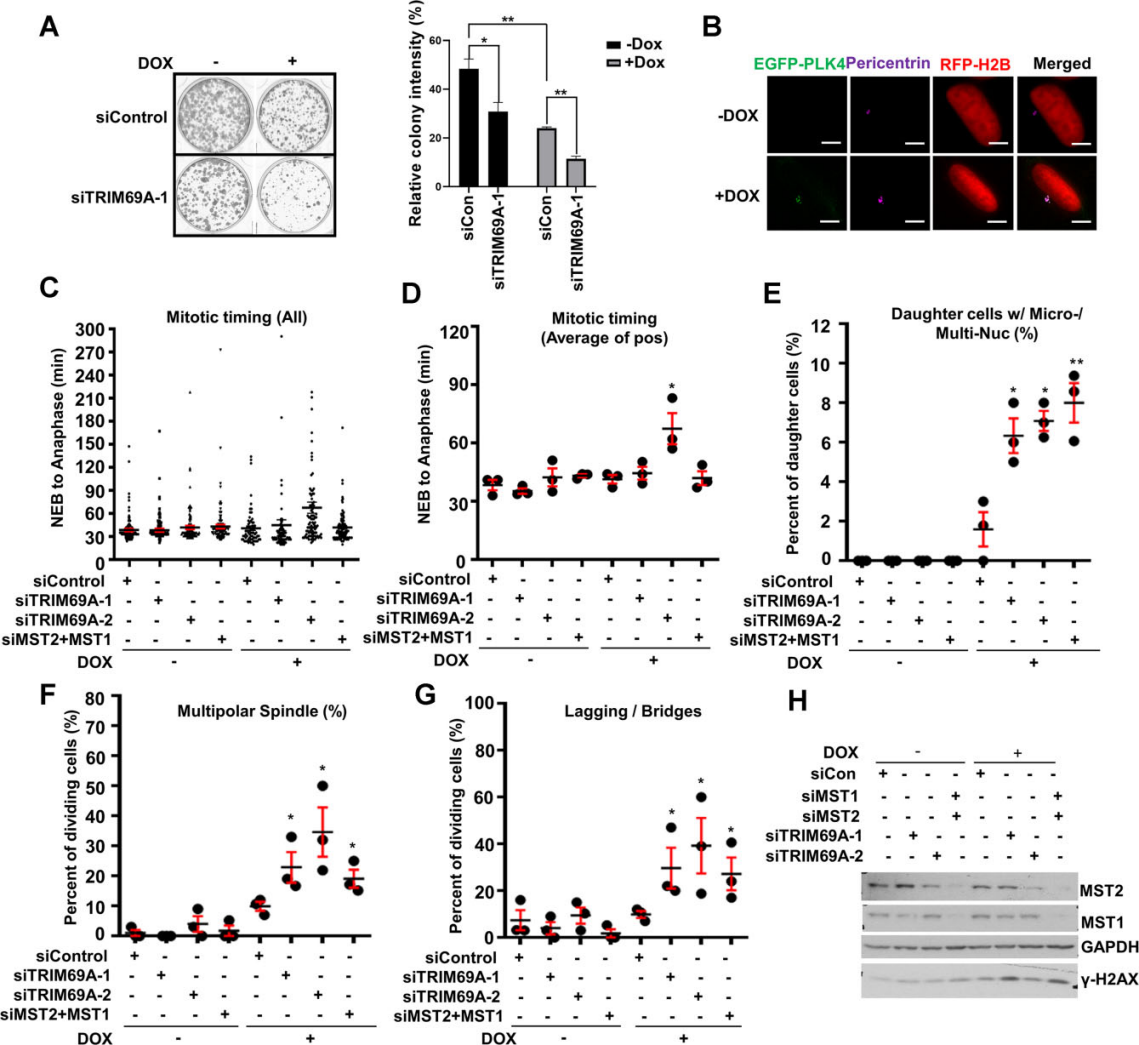
TRIM69A prevents mitotic defects in PLK4-overexpressing cells. (**A**) Effect of TRIM69A siRNA on clonogenic survival of U2OS cells harboring doxycycline (Dox)-inducible GFP-PLK4. The left panel shows representative plates containing stained colonies. On the bar chart, each column represents the mean survival ± SEM of an independent biological replicate. (**B**) Doxycycline-inducible expression of GFP-PLK4 results in increased pericentrin (purple)-staining in a representative U2OS cell stably expressing RFP-H2B. Scale bar represents 5 μm. (**C**) Effect of siRNAs targeting TRIM69A, MST2 and MST1 on mitotic timing of mRFP-H2B-expressing U2OS cells in the presence and absence of excessive PLK4, as determined by time-lapse live cell fluorescence microscopy. The results presented are compiled from three independent experiments. +Dox: siCon, *n* = 69 cells; siTRIM69A1, *n* = 87 cells; siTRIM69A2, *n* = 93 cells; siMST2 + MST1, *n* = 100 cells; –Dox: siCon, *n* = 98; siTRIM69A1, *n* = 101 cells; siTRIM69A2, *n* = 109 cells; siMST2 + MST1, *n* = 116 cells. Data are mean ± SEM. *P ≤ 0.05. Quantification of time-lapse imaging experiments, performed exactly as described in the legend for Figure 7. The results show the effect of siTRIM69A and siMST2/4 treatments on the following measurements: time from NEB to anaphase (**D**); number of daughter cells with micro-/multi-nucleation (**E**); mitotic cells with multipolar spindles (**F**); and mitotic cells with lagging chromosomes and/or anaphase bridges (**G**). Data are mean ± SEM. *P ≤ 0.05, **P ≤ 0.001. (**H**) Immunoblots showing effective depletion of MST2 and MST1 by siRNA treatments in U2OS cells.

In a complementary approach to test the role of TRIM69-MST2 signaling in tolerating excessive centrosomes and spindle multipolarity, we determined the effects of TRIM69 and MST2/1 depletion on mitotic progression of PLK4-overexpressing cells. PLK4 regulates centriole replication and causes centrosome amplification when overexpressed (3,63). Moreover, PLK4 is one of the 20 centrosome amplification (CA) signature genes whose expression is positively related with TRIM69A (Figure 1). We generated human U2OS cells which induce expression of PLK4 from a doxycycline (Dox)-responsive promoter. As expected, Dox-inducible PLK4 expression led to centrosome amplification (evident from pericentrin staining) and a decrease in clonogenic survival (Figure 7A, B). Interestingly, TRIM69-depletion caused increased lethality in PLK4-overexpressing cells (+ Dox) when compared with control (- Dox) cultures (Figure 7A, B). We also performed live-cell imaging to study the effects of TRIM69A and MST2/1 depletion on mitotic progression in the presence and absence of overexpressed PLK4. In uninduced U2OS cells (which rarely have amplified centrosomes), siRNA-mediated ablation of TRIM69A or MST2/1 had no effect on mitotic timing, spindle multi-polarity, micro-nuclei, multi-nucleation, or lagging chromosomes (Figure 7C–G). Interestingly however, in PLK4-overexpressing cells (+ Dox), depletion of TRIM69 or MST2 + MST1 led to increased numbers of multi-polar spindles and other mitotic defects including lagging chromosomes, anaphase bridges and micronuclei (Figure 7C–G). Conversely, ectopically-expressed TRIM69A significantly corrected the spindle multipolarity and other mitotic defects caused by PLK4 induction. TRIM69A overexpression also promoted cell proliferation in cells experiencing PLK4-induced mitotic stress (Supplementary Figure S6). Taken together the results of Figure 7 and Supplementary Figures S5 and S6 show that MST2/1-ablation phenocopies the mitotic defects resulting from TRIM69A-deficiency. We conclude that the TRIM69-MST2/1 signaling axis resolves multipolar spindles and prevents lethal mitoses.

### TRIM69A promotes MT bundling and regulates centrosome clustering through PRC1 and Dynein

**Figure 8.**
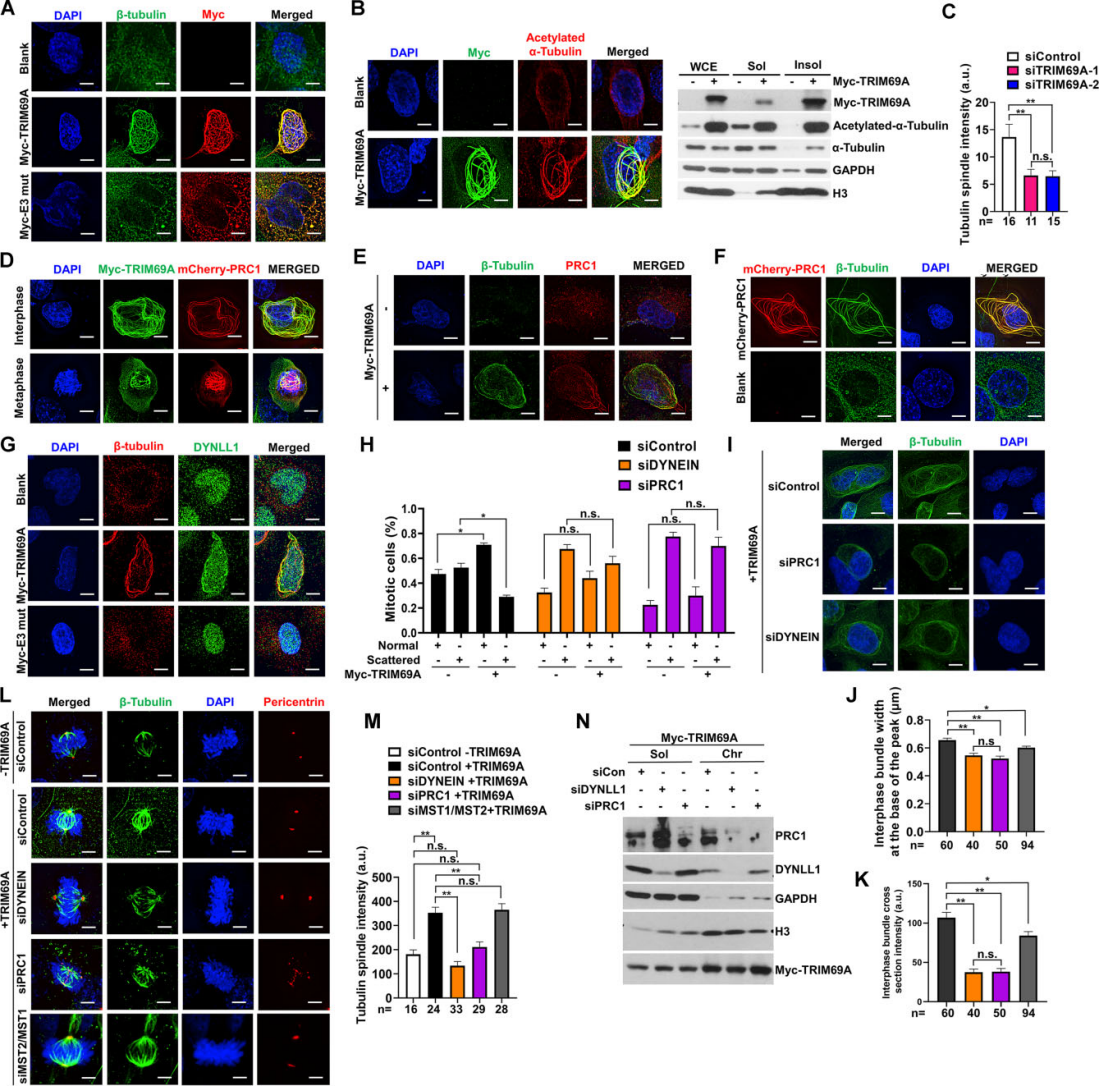
TRIM69A promotes reorganization of microtubules, their associated motors and nucleoporins. (**A**) Representative images showing effect of TRIM69A and TRIM69A E3 mut expression on immunostaining pattern of β-tubulin (green) in H1299 cells. Scale bar represents 5 μm. (**B**) Effect of TRIM69A expression on immunostaining pattern of acetylated α-Tubulin (red) in H1299 cells (left panel). Scale bar represents 5 μm. Immunoblots showing TRIM69A promoted acetylation of α-Tubulin in CSK insoluble fraction (right panel). (**C**) The bar charts illustrate the quantification of tubulin spindle intensity after TRIM69A knockdown, number under bars are number of cells quantified. Data are mean ± standard error of the mean (SEM). ∗∗*P* < 0.0001; n.s., not significant difference. (**D**) Confocal microscopy images of a representative H1299 cell showing co-localization of MYC-TRIM69A (green) with mCherry-PRC1 (red) (Scale bar represents 5 μm). (**E**) Effect of TRIM69A expression on immunostaining of β-tubulin (green) and distribution of endogenous PRC1 (red) in H1299 cells. Scale bar represents 5 μm. (**F**) Confocal microscopy images showing the effect of mCherry-PRC1 (red) overexpression on MT bundling (green) in H1299 cells. (**G**) Effect of TRIM69 expression on immunostaining pattern of DYNLL1 (green) and β-tubulin (red) in H1299 cells. Scale bar represents 5 μm. (**H**) The bar charts illustrate that TRIM69-induced centrosome clustering in paclitaxel-treated cells is inhibited by siRNAs against PRC1 and Dynein. Bars represent mean ± range from two independent experiments. (**I**) Representative images showing effect of PRC1 and DYNEIN knockdown on interphase MT-bundles after TRIM69A overexpression. Immunostaining pattern of β-tubulin (green) is shown. All cells were imaged with the same imaging parameters. Scale bar represents 5 μm. (**J**) The bar charts illustrate the quantification of interphase MT-bundle width at the base of the peak represented in Figure I. *n* is the number of bundles-five bundles per cell. Data are mean ± standard error of the mean (SEM), ∗∗*P* < 0.0001; ∗0.001 > *P* > 0.0001; n.s., not significant. (**K**) The bar charts illustrate the quantification of interphase MT-bundle cross section intensity represented in figure (I). *n* is the number of bundles-five bundles per cell. Data are mean ± standard error of the mean (SEM), ∗∗*p* < 0.0001; ∗0.001 > *p* > 0.0001; n.s., not significant. (**L**) Representative images showing effect of PRC1, DYNEIN and MST2/MST1 knockdown on intensity of individual spindles after TRIM69A overexpression in metaphase H1299 cells. Immunostaining pattern of β-tubulin (green) and Pericentrin (red) is shown. All cells were imaged with the same imaging parameters. Scale bar represents 5 μm. (**M**) The bar charts illustrate the quantification of tubulin spindle intensity represented in (L), numbers under the bars indicate the number of cells quantified. Data are mean ± standard error of the mean (SEM). ∗∗*P* < 0.0001; n.s., not significant differences. (**N**)The immunoblots validate effective downregulation of PRC1 and DYNEIN protein in siRNA experiments.

Our proteomics analyses identified Dynein as a component of the TRIM69 complex (Figure 2C). Therefore, we considered MTs, MT-based motors and MT-bundling proteins such as Dynein and PRC1 (which are implicated in clustering of supernumerary centrosomes) (17,18,64) as candidate mediators of TRIM69A-dependent centrosome clustering. As shown in Figure 8A, B, TRIM69 WT (but not catalytically-dead TRIM69A) induced the formation of MT bundles which contained increased levels of acetylated tubulin, a marker of MT stability (65,66). Conversely, TRIM69A knockdown led to decreased tubulin spindle intensity in metaphase cells (Figure 8C).

Interestingly, TRIM69A co-localized with PRC1 (a MT-binding and bundling protein required for mitotic progression (67)) in both interphase and mitotic cells (Figure 8D). Moreover, TRIM69A promoted localization of PRC1 to bundled MTs (Figure 8E) suggesting that TRIM69A plays a proximal role in PRC1-mediated MT bundling and centrosome clustering. Over-expressed PRC1 fully recapitulated the TRIM69A-induced MT bundling phenotype (Figure 8F), further consistent with participation of TRIM69 and PRC1 in a common pathway that regulates MT reorganization. TRIM69A also promoted localization of the cytoskeletal motor Dynein to bundled MTs (Figure 8G). To test whether bundled MTs are necessary for TRIM69A-dependent centrosome movements, we measured centrosome clustering in cells depleted of PRC1 and Dynein respectively. As shown in Figure 8H, depleting PRC1 or Dynein completely abrogated TRIM69-induced centrosome clustering. Quantification of tubulin-staining revealed that depleting PRC1 or Dynein led to decreased MT bundle width and cross section intensity in interphase (Figure 8I–K) and reduced total tubulin spindle intensity in metaphase (Figure 8L, M). Knockdown efficiencies for PRC1 and DYNEIN are shown in Figure 8N. Although TRIM69 re-localized MST2 to bundled microtubules (Supplementary Figure S7D), MST2 was dispensable for TRIM69A-induced centrosome clustering (Figure 8I–M).

Our mass spectrometry analyses identified nuclear pore proteins NUP205 and NUP133 as components of the TRIM69 complex (Figure 2). Centrosome separation and movement along the nuclear envelope is critically dependent on molecular linkages between nuclear pores and the MT network. Therefore, we determined the effect of TRIM69 on the distribution of tubulin, NUPs, and other potential mediators of centrosome clustering. As shown in Supplementary Figure S7, TRIM69A WT induced co-localization of MST2 with DYNLL1 (Supplementary Figure S7A), CENPF (Supplementary Figure S7B), nuclear pore complex proteins, as evidenced by co-staining with Mab414 (Supplementary Figure S7C) and bundled microtubules (Supplementary Figure S7D). In co-IP experiments, we also detected TRIM69-induced interaction of MST2 with NUPs and MT motors (Supplementary Figure S7E). We conclude that TRIM69A functions upstream of PRC1 in a pathway that leads to MT bundling, reorganizes MT-motor-nucleoporin networks and allows dynein-dependent centrosome clustering.

## DISCUSSION

Here, we identify the protein kinases MST2 and MST1 as new binding partners and effectors of the E3 ubiquitin ligase TRIM69. We demonstrate that TRIM69 stimulates formation of an MST2-PLK1 complex and promotes phosphorylation of MST2 at S15, a known PLK1 site (7). PLK1-mediated MST2 phosphorylation at S15 is necessary for subsequent phosphorylation of NEK2A to dissociate c-NAP1 from daughter centrioles (7). Thus, we provide a new molecular mechanism by which TRIM69 promotes MST2- and PLK1-mediated centrosome disjunction. Importantly, the TRIM69-mediated linker dissolution mechanism defined here is distinct from the growth factor receptor/GRK2-mediated pathway of centrosome disjunction which does not involve PLK1 (68).

A limitation of this work is that we were unable to interrogate the endogenous TRIM69A protein. In numerous experiments with appropriate positive and negative controls, none of the available commercial antibodies (or antibodies that we generated in-house) detected endogenous TRIM69A or TRIM69B. Our results suggest that TRIM69A is present in cells at very low levels and/or that the protein is not very immunogenic. Therefore, out of necessity we have studied tagged forms of TRIM69 in our experiments. Historically, studies with ectopically-expressed proteins have been critical for generating mechanisms of action and paradigms for E3 ligase signaling and genome maintenance. Some landmark studies in the centrosome field have relied primarily on ectopically-expressed proteins to define biochemical interactions and signaling mechanisms that mediate centrosome disjunction (7,69). Nevertheless, in the future when better reagents are available, it will be necessary to validate roles of endogenous TRIM69 and other factors in regulating centrosome biology.

In addition to promoting centrosome disjunction, we show that TRIM69 stimulates centrosome clustering, both after taxol treatment (which promotes multi-polar mitoses) and following PLK4 overexpression (which promotes centrosome replication and amplification). There is no evidence that the canonical PLK1/MST2-mediated centrosome disjunction pathway can explain centrosome clustering activity of TRIM69-MST2. Therefore, we must consider possible mechanisms whereby TRIM69-MST2 might promote centrosome clustering.

An elegant genetic screen for mediators of centrosome clustering in *Drosophila* cells yielded three categories of genes: (1) participants in the Spindle Assembly Checkpoint (SAC); (11) genes encoding MT-associated proteins and motors with roles in spindle focusing and (111) genes involved in cell adhesion-based centrosome movement (64,70). While we have not examined connections between TRIM69 and the SAC or cell adhesion, TRIM69A certainly has hallmarks of centrosome-clustering genes in category #2, i.e. MT-associated proteins and motors. For example, we show that TRIM69 induces robust MT bundling, and also forms complexes with MT motors (DYNEIN), MT-associated proteins (PRC1) and nucleoporins that tether centrosomes to the nucleus and regulate centrosome movements.

Clustering of supernumerary centrosomes is often associated with MT stabilization and involves the same mediators that bundle MTs into bipolar spindle arrays in normal cells (71). Forces responsible for centrosome clustering can be generated by MT-bundling complexes that reside where anti-parallel MTs overlap (59). For example, the MT-bundling protein PRC1, crosslinks microtubules into antiparallel arrays and cooperates with motor proteins to control the dynamics and size of bundled regions (72). Thus PRC1 is necessary for central spindle formation (73) and kinetochore tension in metaphase (74,75). Moreover, PRC1 facilitates clustering of supernumerary centrosomes in metaphase, which prevents spindle multipolarity (18).

In addition to MT bundling and stabilization, MT motors and their connections with the nuclear envelope are important for centrosome clustering and separation. In normal cells, dynein stabilizes interphase microtubule arrays and determines centrosome position (76). Dynein mediates attachment and migration of centrosomes along the nuclear envelope during interphase/prophase and facilitates attachment of centrosomes to spindle poles in mitosis (77). In cells with supernumerary centrosomes, dynein helps coalesce the excess centrosomes into pseudo-bipolar spindles. Interference with dynein localization promotes centrosome scattering and multipolarity (17). During G2 and prophase, MTs emanating from centrosomes connect to the nuclear envelope via two independent and cooperating dynein-mediated mechanisms: (i) nuclear pore proteins RanBP2 and NUP358 at the cytoplasmic side of the nuclear membrane recruit BICD2 which tethers dynein/dynactin to NPCs. (ii) The nucleoporin NUP133 interacts with CENPF in G2/M. In turn, CENPF recruits NudE/NuDEL which interact with dynein (78). These dynein-mediated connections between the nuclear pores and the MT networks help drive centrosome separation and critically maintain centrosome association with the nuclear envelope (78). Depletion of nucleoporins leads to supernumerary centrosomes and multipolar spindles (79) further consistent with a role for nuclear pore complexes in centrosome clustering. Taken together, our results suggest that PRC1 and DYNEIN, together with TRIM69 play important roles in facilitating formation of stable bundled MT networks and regulating their associated proteins, including NUPs, to promote centrosome clustering and centrosome separation.

Here, we propose that TRIM69A promotes association of PRC1 with antiparallel MT bundles, including those that connect the extra centrosome (s) with the bipolar spindle. Stability of these overlap regions supports extra centrosome coalescence with the centrosomes from the bipolar spindle by zippering up these overlaps with the spindle. Likewise, TRIM69A promotes Dynein association with microtubules and this likely accounts for the minus end directed pulling of an extra centrosome towards a centrosome of the bipolar spindle. Our finding that PRC1 and Dynein depletions have similar effects on spindle multipolarity suggest that these two microtubule-associated proteins act cooperatively in this process.

In our study, a catalytically-dead TRIM69A mutant was also inactive for MST2-relocalization, centrosome disjunction, and centrosome clustering activities. Further studies are necessary to identify the putative TRIM69A substrate(s) whose ubiquitination is necessary for regulating and interacting MST2 and centrosome dynamics. To address this issue, we have performed an unbiased screen for TRIM69A-induced ubiquitination events. As shown in Supplementary Figure S5F, we identified several proteins (including nucleoporins) that were ubiquitinated in a TRIM69-inducible manner and which represent potential mediators of centrosome disjunction or clustering. We are also considering the alternative hypothesis that TRIM69A mediates MST2 localization and regulation of centrosome function in a manner that is separable from its E3 ubiquitin ligase activity. The E3 ligase-inactive TRIM69A mutant used in this study harbors substitutions in the RING domain. In addition to abrogating E3 ligase activity, RING domain mutations could also prevent protein-protein interactions. Some RING finger-containing E3 ubiquitin ligases have important biological activities that are independent of their catalytic activities. For example the E3 ubiquitin ligase RAD18 mono-ubiquitinates PCNA to promote Trans-Lesion Synthesis at stalled DNA replication forks. However, RAD18 also binds directly to DNA polymerase Eta (Polη), and acts as a molecular chaperone that deposits Polη at DNA replication forks independent of its PCNA ubiquitination activity (80,81). RAD18 also acts as a molecular chaperone for the HR factor RAD51B (82) independently of its catalytic activity. Based on this precedent, TRIM69A functions in centrosome disjunction and clustering could be mediated solely through chaperoning and re-localization of MST2.

TRIM69 belongs to a family of SUMO-Targeted Ubiquitin Ligases (STUBLs) which associate with SUMOylated target proteins (83,84). Therefore, it will also be important to identify the putative SUMOylated proteins that bind and recruit TRIM69A. Appropriate SUMOylation/de-SUMOylation is important for centromere-MT attachment and hundreds of key mitotic factors are SUMOylated (84) and represent potential receptors for recruiting TRIM69A. Moreover, our proteomics experiments identified nucleoporins as major binding partners of TRIM69 and MST2. The nucleoporin NUP358 is an E3 SUMO ligase complex (85) and is localized to the centrosomes (86). Therefore, NUP358 or its SUMOylated substrates might be important for recruiting TRIM69A to the centrosomes.

Many of the tripartite motif (TRIM) proteins have roles in cell cycle phase transitions, particularly mitotic progression, and are implicated in human diseases including cancer (87). Interestingly, the E3 ligase TRIM37 prevents the formation of aberrant centrosomal protein assemblies that function as extra MTOCs and cause segregation defects (88,89). Our work identifies new cancer-relevant mitotic mechanisms for TRIM69, thereby expanding our understanding of biological roles of the TRIM family members. Song et al recently reported that TRIM69 binds and stabilizes MTs in interferon-stimulated myeloid cells to limit viral spread (90). Given the diverse cellular functions of microtubules we consider it likely that TRIM69 influences additional MT-dependent processes.

The molecular and biochemical roles we have defined for TRIM69A in centrosome function fully explain why high expression of TRIM69 mRNA is correlated so highly with the CA20 gene signature in basal breast cancers (Figure 1). It is interesting to note that those CA20/TRIM69-high basal cancers also tend to have p53 mutations, and HR-deficiency, tumorigenic features which are conducive to centrosome amplification (91,92). Similar to TRIM69, other MT bundling and centrosome-clustering proteins are also typically overexpressed in cancer (20,71). We suggest that TRIM69A expression promotes centrosome clustering and viable cell divisions and is an enabling characteristic of cancer cells harboring supernumerary centrosomes.

## Supplementary Material

gkad766_Supplemental_File

## Data Availability

The data underlying this article are available in the PRIDE archive at https://www.ebi.ac.uk/pride/archive, and can be accessed under Accession Code PXD042098.
