# Supplementary material for: The TRIM69-MST2 signaling axis regulates centrosome dynamics and chromosome segregation
Source: Nucleic Acids Res. 2023 Sep 22;51(19):10568–89. doi: 10.1093/nar/gkad766 (PMC10602929; doi:10.1093/nar/gkad766)
Supplement: gkad766_Supplemental_File [file gkad766_supplemental_file.pdf]

The TRIM69-MST2 Signaling Axis Regulates Centrosome Dynamics and Chromosome Segregation

Yilin Wang[1], Patrik Risteski[3], Yang Yang[1,2], Huan Chen[4], Gaith Droby[1,5], Andrea Walens[2], Deepika Jayaprakash[1], Melissa Troester[6], Laura Herring[7], Jonathan Chernoff[8], Iva M. Tolić[3], Jessica Bowser[1], Cyrus Vaziri[1,2*,#]

[1] Department of Pathology and Laboratory Medicine, University of North Carolina, Chapel Hill, NC 27599, USA

[2] Lineberger Comprehensive Cancer Center, University of North Carolina, Chapel Hill, NC 27599, USA

[3] Division of Molecular Biology, Ruđer Boskovic Institute, Bijenicka cesta 54, 10000 Zagreb, Croatia

[4] Joint Center for Single Cell Biology, School of Agriculture and Biology, Shanghai Jiao Tong University, Shanghai 200240, China

[5] Curriculum in Genetics and Molecular Biology, University of North Carolina, Chapel Hill, NC 27599, USA

[6] Department of Epidemiology, Gillings School of Global Public Health and UNC Lineberger Comprehensive Cancer Center, University of North Carolina, Chapel Hill, NC 27599, USA

[7] Department of Pharmacology, UNC Proteomics Core Facility, University of North Carolina, Chapel Hill, NC 27599, USA

[8] Fox Chase Cancer Center, Philadelphia, PA 19111, USA

*To whom correspondence should be addressed. Tel: +1(919)843-9639; Email: cyrus_vaziri@med.unc.edu

Content

Figures S1-S7 **pp. S2-S8**

Table S1 **pp. S9**

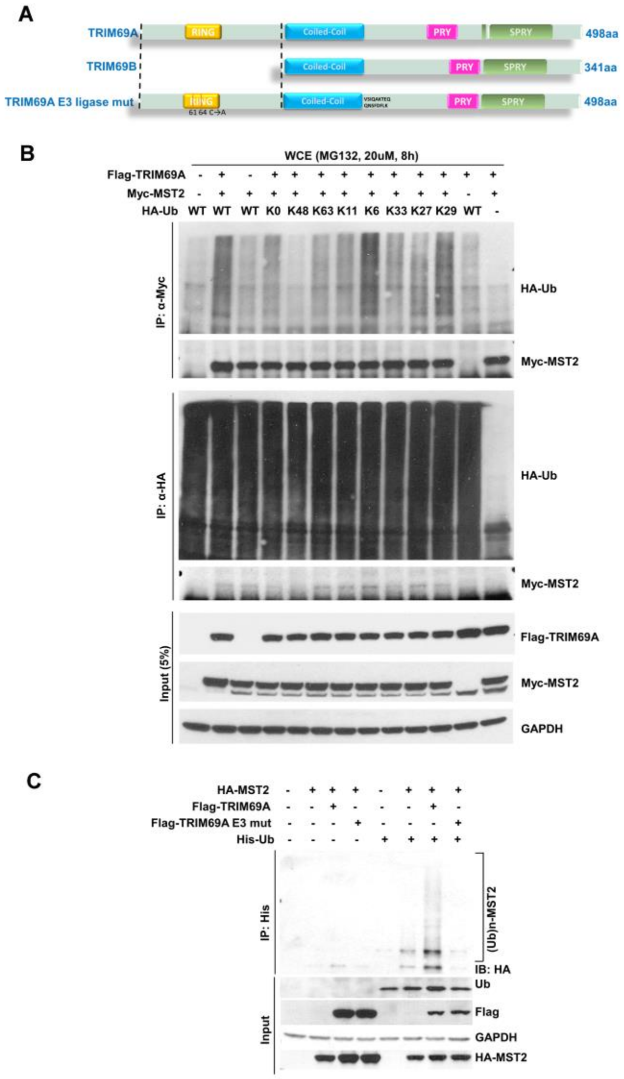

Figure S1. (**A**) Schematic presentation of TRIM69 domains, isoforms, and mutants used here. (**B**) Immunoblots showing TRIM69A promotes K6- and K29-linked uniqitination of MST2. Ubiquitination and immunoblotting were performed with the indicated antibodies. (**C**) Immunoblots showing ubiquitination of MST2 without MG132.

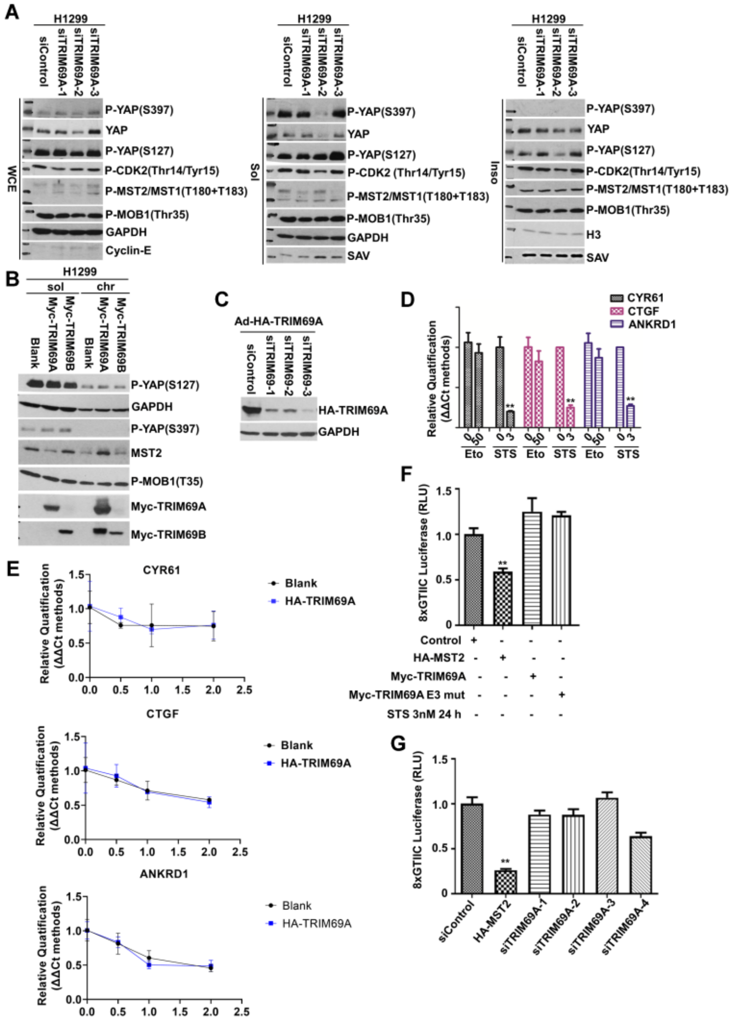

Figure S2. The TRIM69-MST2 signaling axis does not affect the Hippo pathway. (**A**) Immunoblots showing that Hippo pathway proteins are unaffected by treatment with TRIM69A-directed siRNAs in H1299 cells. (**B**) Immunoblots showing that Hippo pathway proteins are unaffected by TRIM69 overexpression in H1299 cells. (**C**) Immunoblots showing effective ablation of TRIM69A by multiple independent siRNAs in H1299 cells. (**D**) qRT-PCR analysis of CYR61, CTGF and ANKRD1 mRNA expression in HEK293T cells treated with Ethylene Oxide (ETO) or Staurosporine (STS). P-values were calculated using Student's t-test. Data are mean ± SEM. **P < 0.01. (**E**) qRT-PCR analysis showing effect of TRIM69A expression and STS treatment on expression levels of CYR61, CTGF and ANKRD1 mRNAs in HEK293T cells. (**F**) Effect of TRIM69A and TRIM69A E3 mut on expression of a luciferase reporter gene driven by a YAP1-responsive (8xGTIIC) promoter in HEK293T cells. P-values were calculated using Student's t-test. Data are mean ± SEM. (**G**) Effect of TRIM69 siRNA treatments on YAP1-responsive 8xGTIIC-driven luciferase reporter activity in HEK293T cells. P-values were calculated using Student's t-test. Data are mean ± SEM.

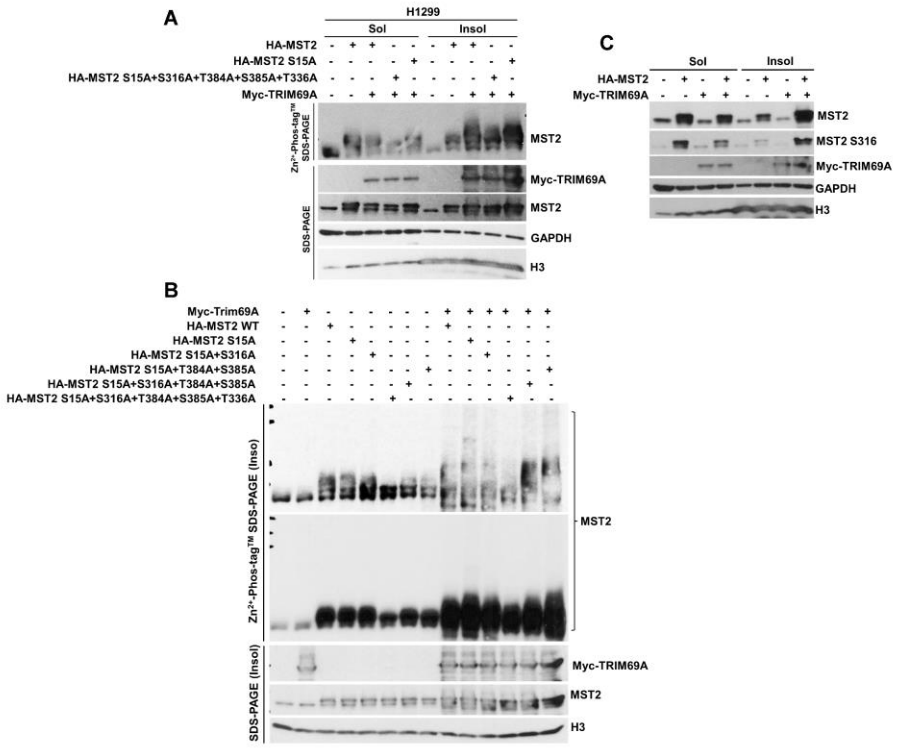

Figure S3. Defining TRIM69A-induced MST2 phosphorylation sites. (**A**)-(**B**) Results of phos-tag phosphate-affinity gel electrophoresis and immunoblotting experiments showing mobility shifts of MST2 WT and MST2 mutant proteins harboring individual or combinatorial alanine substitutions in the indicated phosphorylation sites. (**C**) Results of SDS-PAGE and immunoblotting experiments using a MST2 phospho-specific antibody and showing TRIM69A-induced phosphorylation of MST2 at S316.

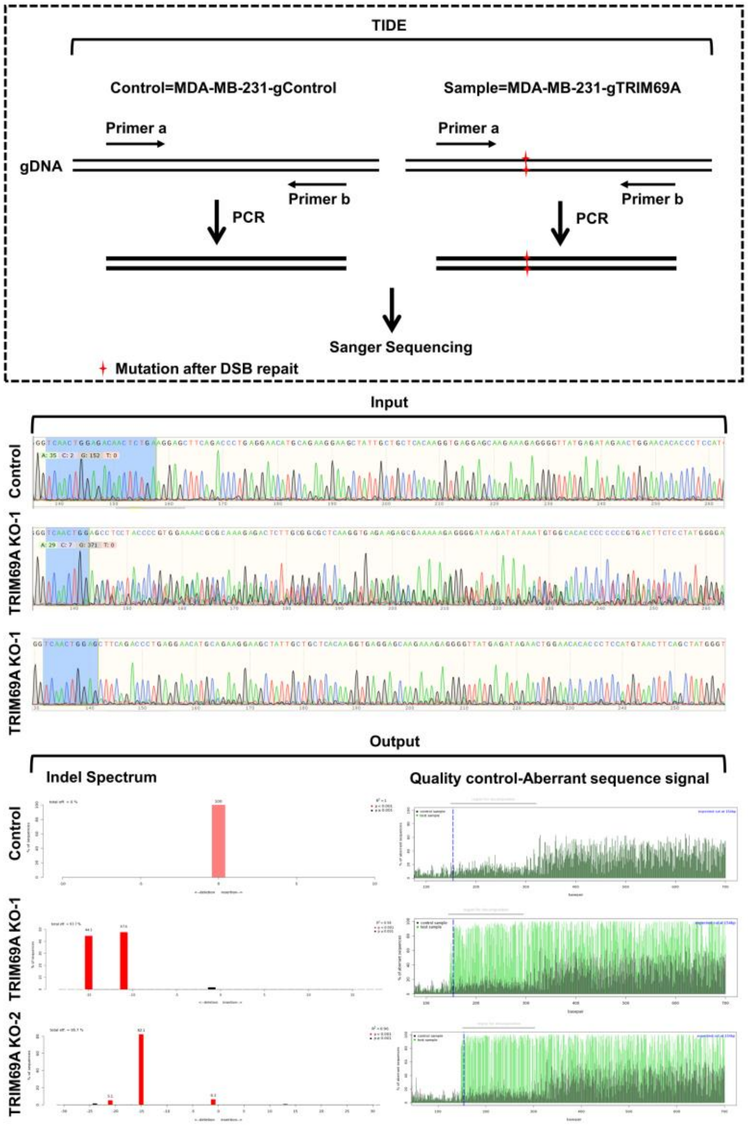

Figure S4. TIDE assay showing disruption of the*TRIM69* gene in MDA-MB-231 cells. Sequencing results are shown for three different cell lines.

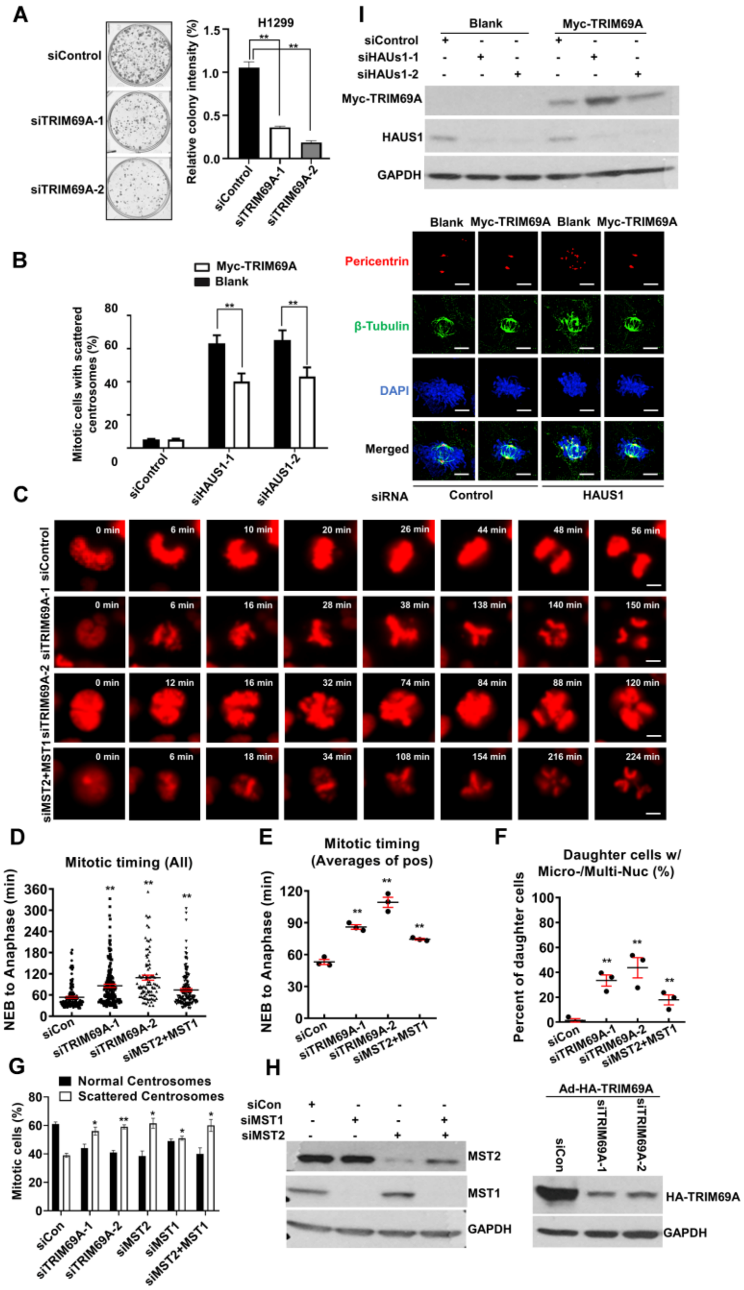

Figure S5. TRIM69A and MST2 prevent mitotic defects and limit centrosome scattering in paclitaxel-treated cells. (**A**) Treatment with TRIM69 siRNA reduces viability of H1299 as determined by colony formation assays. The bar chart shows quantification of data from clonogenic survival assays. Each data point represents the mean of 3 replicate determinations ± SEM. **P ≤ 0.001. (**B**) Ectopically-expressed MYC-TRIM69A stimulates centrosome clustering in HAUS1 siRNA-treated H1299 cells. The bar chart shows quantification of the cells with scattered centrosomes. Bars represent the mean number of cells with scattered centrosomes and the error bars show the range. The data were derived from 2 independent experiments. Confocal microscopy images showing representative cells quantified in Sup. Fig. 4B. Scale = 5 µm. (**C**) Time-lapse live cell fluorescence microscopy showing effect of siRNAs targeting TRIM69A, MST2 and MST1 on mitotic progression of mRFP-H2B-expressing H1299 cells in the presence of paclitaxel. Images were acquired every 2 min for 24 h. The panels show representative images of individual nuclei from different treatment conditions

undergoing mitosis. Time after NEB is indicated. Scale = 5 µm. (**D**) Quantification of experiments represented in C. For each transfected siRNA, the time spent in mitosis was measured from NEBD to anaphase. The results presented are compiled from three independent experiments. siCon, n = 170 cells; siTRIM69A, n =170 cells; siTRIM69A-1, n = 91 cells; siMST2+MST1, n =172 cells. Horizontal lines represent means and red error bars indicate the ± standard error of the mean (SEM) of three independent experiments. (**E – F**) For each treatment condition, the combined data in panel D were derived from three independent live cell movies captured from separate fields. In panel E each data point represents the mean duration of NEB-Anaphase for each individual field ± SEM. In panel F each data point represents the mean number of daughter cells containing micro-nuclei and/or multiple nuclei for each individual field ± SEM. *P ≤ 0.05, **P ≤ 0.001. (**G**) Effect of TRIM69, MST2, and MST1 siRNAs on centrosome scattering in paclitaxel-treated H1299 cells. Cells were visualized by immunofluorescence microscopy and 100 mitotic cells were analyzed for each treatment condition. Bars represent the mean numbers of scattered and normal centrosomes ± range from 2 independent experiments. (**H**) Immunoblotting experiments to validate siRNAs used to downregulate TRIM69 and MST2/1 in A-G. (**I**) Immunoblots validate effective downregulation of HAUS1 protein in siRNA experiments.

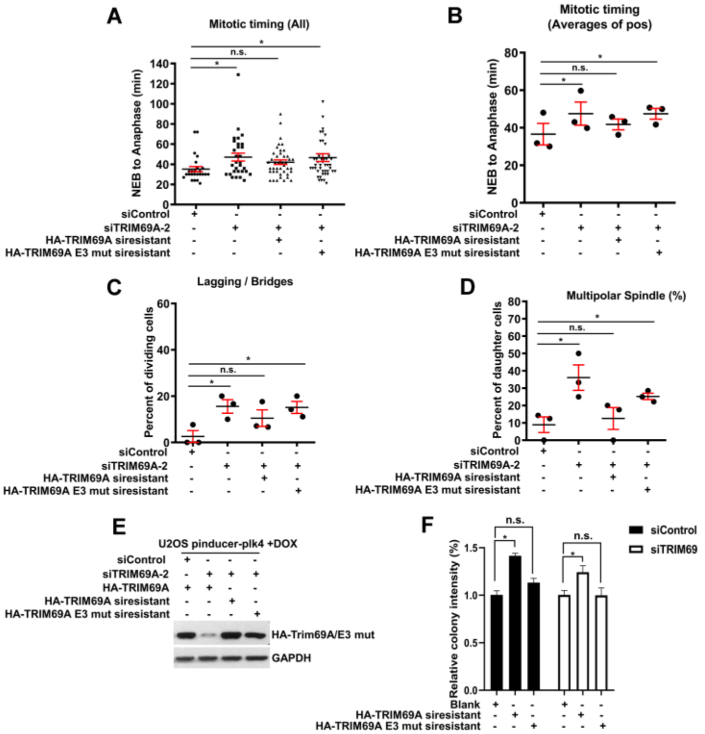

Figure S6. Ectopically-expressed TRIM69A rescues mitotic defects of PLK4-overexpressing cells. (**A**) Effect of TRIM69A WT or TRIM69A E3 mut on mitotic timing of mRFP-H2B-expressing U2OS cells in the presence of excessive PLK4, as determined by time-lapse live cell fluorescence microscopy. The results presented are compiled from 3 independent experiments. siCon, n = 35 cells; siTRIM69A-2, n =36 cells; siTRIM69A-2+HA-TRIM69A (siRNA-resistant), n =36 cells; siTRIM69A-2+HA-TRIM69A E3 mut (siRNA-resistant), n=49 cells. Data are mean ± SEM. *P ≤ 0.05. Quantification of time-lapse imaging experiments were performed exactly as described in the legend for Supplementary Fig. 6. The results show the effect of TRIM69A expression on the following measurements: time from NEB to anaphase (**B**); mitotic cells with lagging chromosomes and/or anaphase bridges (**C**); mitotic cells with multipolar spindles (**D**). Data are mean ± SEM. *P ≤ 0.05. (**E**) Immunoblots showing effective depletion of TRIM69A by siRNA treatment and reconstitution of siRNA-resistant TRIM69A or TRIM69A E3 mut in U2OS cells. (**F**) Effect of TRIM69A or TRIM69A E3 mut overexpression on clonogenic survival of U2OS cells harboring doxycycline (Dox)-inducible GFP-PLK4. Each column of the bar chart represents the mean survival ± SEM of an independent biological replicate.

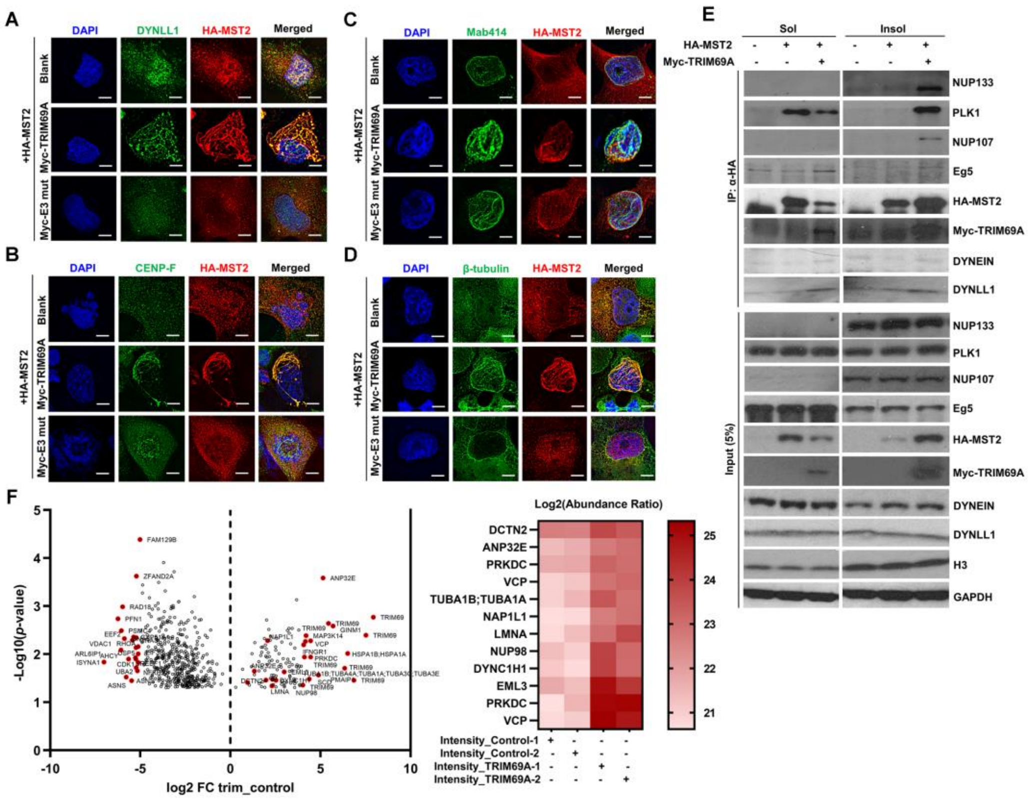

Figure S7. (**A**) Effect of TRIM69 expression on immunostaining pattern of DYNLL1 (green) and HA-MST2 (red) in H1299 cells. Scale bar represents 5 µm. (**B**) Effect of TRIM69 expression on immunostaining pattern of CENP-F(green) and HA-MST2 (red) in H1299 cells. Scale bar represents 5 µm. (**C**) Effect of TRIM69 expression on immunostaining pattern of NUP107-Mab414 (green) and HA-MST2 (red) in H1299 cells. Scale bar represents 5 µm. (**D**) Effect of TRIM69 expression on immunostaining pattern of β-tubulin (green) and HA-MST2 (red) in H1299 cells. Scale bar represents 5 µm. (**E**) TRIM69A promotes formation of nucleoporin-containing protein complexes. (**F**) Identification of TRIM69A substrates using the KGG IP coupled to LC/MS/MS. Student's t-test was performed on TRIM69A vs Control. P-value of <0.5 or lower means significant.

Supplementary Table S1. List of primer probe set, siRNA and sgRNA sequences.

Primer Name	Primer Sequences (5'-3')
**Cloning**	
TRIM69A-Y2H-F	GGGGACAAGTTTGTACAAAAAAGCAGGCTTCATGGAGGTATC CACCAACCCCTCC
TRIM69A-Y2H-R	CGGGACCACTTTGTACAAGAAAGCTGGGTCTTACTGTGGATG TAAGATGTGC
TRIM69B-Y2H-F	GGGGACAAGTTTGTACAAAAAAGCAGGCTTCATGGAGGAGG AGCTTGCCATC
TRIM69B-Y2H-R	CGGGACCACTTTGTACAAGAAAGCTGGGTCTTAGTCTTAAAA GCCAGAATCC
MST1-Y2H-F	GGGGACAAGTTTGTACAAAAAAGCAGGCTTCATGGAGACGG TACAGCTGAGG
MST1-Y2H-R	CGGGACCACTTTGTACAAGAAAGCTGGGTCTCAGAAGTTTTG TTGCCGTCTC
MST2-Y2H-F	GGGGACAAGTTTGTACAAAAAAGCAGGCTTCATGGAGCAGC CGCCGGCGC
MST2-Y2H-R	CGGGACCACTTTGTACAAGAAAGCTGGGTCTCAAAAGTTTTG CTGCCTTCTTTTCTTTG
pcDNA3-HA-TRIM69A-F	GTTTAGTGAACCGTCAGATCGCCACCATGGCCTACCCATAT
pcDNA3-HA-TRIM69A-R	GAACCGCGGGCCCTCTAGACCTTACTGTGGATGTAAGATGT GCAATG
pCMV-Myc-TRIM69A E3 mut-F	GCTGAAGCCGCTATCCAAGACTTTTGGAGGCTGC
pCMV-Myc-TRIM69A E3 mut-R	GAAGTTGTGGCCACAGCTTAGCATCAG
pcDNA3-Flag-TRIM69A-F	CGGGATCCatgGATTACAAGGATGACGACGATAAGATGGAGG TATCCACCAACCCCTCC
pcDNA3-Flag-TRIM69A-R	CGGAATTCTTACTGTGGATGTAAGATGTGCAATGGTTC
pcDNA3-Flag-TRIM69A E3 mut-F	GCTGAAGCCGCTATCCAAGACTTTTGGAGGCTGC
pcDNA3-Flag-TRIM69A E3 mut-R	GAAGTTGTGGCCACAGCTTAGCATCAG
pcDNA3-HA-TRIM69A siresistant-F	CATCCAAGGTAGTAATCCAAGATATTACTATG
pcDNA3-HA-TRIM69A siresistant-R	GTAGGTGGGTGATTGAATCATTCATTTC
pcDNA3-HA-TRIM69A E3 mut siresistant-F	GCTGAAGCCGCTATCCAAGACTTTTGGAGGCTGC
pcDNA3-HA-TRIM69A E3 mut siresistant-R	GAAGTTGTGGCCACAGCTTAGCATCAG
pinducer-eGFP-PLK4-F	GTTTAGTGAACCGTCAGATCATGGTGAGCAAGGGCGAGGAG C
pinducer-eGFP-PLK4-R	GAACCGCGGGCCCTCTAGACCTTATCTAGATCCGGTGGATC CTTTGTCATC
pinducer-eGFP-CENTRIN2-F	GTTTAGTGAACCGTCAGATCATGGTGAGCAAGGGCGAGGAG C
pinducer-eGFP-CENTRIN2-R	GAACCGCGGGCCCTCTAGACCTTATCTAGATCCGGTGGATC CCGGG
pcDNA-HA-MST2-S15-F	CTGGCAGAAGACAGTTTGACTAAG
pcDNA-HA-MST2-S15-R	CTTTTTTAGTTTACTCTTAGGCGCCG
pcDNA-HA-MST2-S385-F	CAACCGCACCACAAGTACAAAGAC

pcDNA-HA-MST2-S385-R	CATTTCTTTTCATAGTTCCATCTTCTTC
pcDNA-HA-MST2-S316-F	GAAAATGCAGATGAAGATGAGCTGGATTC
pcDNA-HA-MST2-S316-R	TTCTTCCTCTTCCAATTCTCGTTGCTG
pcDNA-HA-MST2-T384-F	GCAGCATCACCACAAGTACAAAGAC
pcDNA-HA-MST2-T384-R	ATTTCTTTTCATAGTTCCATCTTCTTCTTCC
pcDNA-HA-MST2-T336-F	GGCGCAATGCGGGCCACAAG
pcDNA-HA-MST2-T336-R	CACACTCTCCACACTAGTCTTCACCATG
pcDNA3-NEK2A-Flag-F	CGCGGATCCATGCCTTCCCGGGCTGAGGAC
pcDNA3-NEK2A-Flag-F	CCGCTCGAGCTATTTATCATCATCATCTTTATAATCGCGCATG CCCAGGATCTGTCTG
Genotyping primer for TRIM69A sgRNA3 F	GAACTATGATACAGGGGAAGGGGAAG
Genotyping primer for TRIM69A sgRNA3 R	AGGTACCATTTAGTGTGTAGATAATATATGTTAGGC
Genotyping primer for TRIM69A sgRNA9 F	CCACCAACCCCTCCTCCAACATC
Genotyping primer for TRIM69A sgRNA9 R	CTTGCATTGAAAGCAGATCAGTTTCCC
lentiCRISPR v2 sgRNA verification primer	GTAGGCGTGTACGGTGGGAGG
**sgRNA**	
TRIM69A sgRNA3	GTGGAAAGGACGAAACACCGTCAACTGGAGACAACTCTGAG TTTTAGAGCTAGAAATAGCAAGTTAAAATAAGG
TRIM69A sgRNA9	GTGGAAAGGACGAAACACCGCTTTTGGAGGCTGCAAGCAAG TTTTAGAGCTAGAAATAGCAAGTTAAAATAAGG
**qRT-PCR**	
CYR61 F	AAGAAACCCGGATTTGTGAG
CYR61 R	GCTGCATTTCTTGCCCTTT
CTGF F	CTCCTGCAGGCTAGAGAAGC
CTGF R	GATGCACTTTTTGCCCTTCTT
ANKRD1 F	TTTGGCAATTGTGGAGAAGTTA
ANKRD1 R	AAACATCCAGGTTTCCTCCA
β-actin F	AACTACCTTCAACTCCATCA
β-actin R	GAGCAATGATCTTGATCTTCA
GAPDH F	ATGGGGAAGGTGAAGGTCG
GAPDH R	GGGGTCATTGATGGCAACAATA
**siRNA**	
siHAUS1-1	Ambion Cat. #AM16708 siRNA ID: 148017
siHAUS1-2	Ambion Cat. #AM16708 siRNA ID: 148018
siControl	UAGCGACUAAACACAUCAAUU
siTRIM69A-1	GGGAAGUAGAAGUAGCAAAUU
siTRIM69A-2	CCUCUAAAGUGGUCAUACAUU
siTRIM69A-3	AGUUGGUAGAGAAGAUUAAGAUU
siTRIM69A-4	GCGACUAUGUUGAAAUGAAUGUU
siTRIM69A-5	AGAUGCUAUGUCAGUAUAACAUU

siMST2	Dharmacon Cat. #M-004874-02-0010 siGENOME SMARTpool
siMST1	GGGCACUGUCCGAGUAGCAUU
siPRC1	Dharmacon Cat. #L-019491-00-0005
siDYNLL1	Dharmacon Cat. #L-005281-00-0005
